# Unraveling metabolic patterns and molecular mechanisms underlying storability in sugar beet

**DOI:** 10.1186/s12870-022-03784-6

**Published:** 2022-09-09

**Authors:** Anna-Lena Gippert, Silvia Madritsch, Patrick Woryna, Sandra Otte, Martina Mayrhofer, Herbert Eigner, Adriana Garibay-Hernández, John C. D’Auria, Eva M. Molin, Hans-Peter Mock

**Affiliations:** 1 IPK Leibniz Institute of Plant Genetics and Crop Plant Research, Gatersleben, Germany; 2 AIT Austrian Institute of Technology GmbH, Center for Health & Bioresources, Tulln, Austria; 3 Strube Research GmbH & Co. KG, Söllingen, Germany; 4 AGRANA Research & Innovation Center GmbH, Tulln, Austria

**Keywords:** Sugar beet, Storability, Transcriptomics, Metabolomics, Molecular mechanisms, Integrative analysis

## Abstract

**Background:**

Sugar beet is an important crop for sugar production. Sugar beet roots are stored up to several weeks post-harvest waiting for processing in the sugar factories. During this time, sucrose loss and invert sugar accumulation decreases the final yield and processing quality. To improve storability, more information about post-harvest metabolism is required. We investigated primary and secondary metabolites of six sugar beet varieties during storage. Based on their variety-specific sucrose loss, three storage classes representing well, moderate, and bad storability were compared. Furthermore, metabolic data were visualized together with transcriptome data to identify potential mechanisms involved in the storage process.

**Results:**

We found that sugar beet varieties that performed well during storage have higher pools of 15 free amino acids which were already observable at harvest. This storage class-specific feature is visible at harvest as well as after 13 weeks of storage. The profile of most of the detected organic acids and semi-polar metabolites changed during storage. Only pyroglutamic acid and two semi-polar metabolites, including ferulic acid, show higher levels in well storable varieties before and/or after 13 weeks of storage. The combinatorial OMICs approach revealed that well storable varieties had increased downregulation of genes involved in amino acid degradation before and after 13 weeks of storage. Furthermore, we found that most of the differentially genes involved in protein degradation were downregulated in well storable varieties at both timepoints, before and after 13 weeks of storage.

**Conclusions:**

Our results indicate that increased levels of 15 free amino acids, pyroglutamic acid and two semi-polar compounds, including ferulic acid, were associated with a better storability of sugar beet taproots. Predictive metabolic patterns were already apparent at harvest. With respect to elongated storage, we highlighted the role of free amino acids in the taproot. Using complementary transcriptomic data, we could identify potential underlying mechanisms of sugar beet storability. These include the downregulation of genes for amino acid degradation and metabolism as well as a suppressed proteolysis in the well storable varieties.

**Supplementary Information:**

The online version contains supplementary material available at 10.1186/s12870-022-03784-6.

## Background

Sugar beet is an important crop for sugar production since it accumulates high sucrose concentrations in the root and its yield has continuously increased over the past decades [1]. However, breeding strategies that exclusively consider high yield potential have reached a physiological bottleneck demanding alternative improvements [2]. Besides cultivation and harvest [3, 4], the post-harvest situation can also reduce sucrose concentration, especially during sugar beet storage. Lately, sucrose loss during storage has become more important since an increasing centralization of sugar factories in Europe demands a prolongation of storage of harvested beet roots for up to 90 days [5]. During this time, beet roots maintain an active metabolism that utilizes stored sucrose reserves lowering industrial yields [6]. Furthermore, since sucrose metabolism is accompanied by invert sugar accumulation, storage reduces the beet processing quality [7]. Invert sugars and other non-sucrose compounds, like sodium, potassium, and amino nitrogen, in the beet root are considered as impurities, because they impede industrial sucrose extraction [8].

One of the major causes of post-harvest sucrose loss is respiration, an oxidative process which consumes sucrose to provide metabolic energy and carbon substrates for the sugar beet’s metabolism [9]. Different factors can influence the respiration rate enhancing sucrose consumption and invert sugar production, and thereby affecting the root’s physical integrity and its overall storage capacity. Among these factors, two important ones are the storage condition, including temperature, humidity and duration, and the harvesting practice. For example, beet root damage at harvest leads to increased pathogen infestation and higher invert sugar content [10, 11]. In addition to biotic stressors [12, 13], abiotic stress such as drought during beet cultivation further increased sucrose loss and the accumulation of impurities [14]. Genetic factors also impact the storage capacity of sugar beet. Campbell and Klotz (2014) revealed that the effect of the variety was observed mostly during stressful cultivation and manifested itself through a variety-specific pathogen infestation and abnormal carbohydrate metabolism [15]. They also found that the influence of the variety on storage losses was up to 12%, highlighting its relevance for sugar beet variety selection. Schnepel and Hoffmann (2016) further investigated the effects of genotypic variation during storage by cultivating eighteen sugar beet varieties at sites in Germany and Spain, the latter one characterized by increased heat and drought stress [16]. They found that varieties with a higher marc content lost less sucrose during storage, correlating with a higher resistance to pathogens. Other indicators of storability like the invert sugar content after stressful cultivation or the sugar concentration in dry matter were however independent of the variety. Recently, Madritsch et al. (2020) reported that varieties with low sucrose loss during storage (which were defined as well storable varieties) displayed more parenchyma cells, a smaller cell area, and a thinner periderm [17]. Furthermore, they found more than 900 differentially expressed genes between the well and the rather badly storable varieties, including genes related to defense, carbohydrate, and phenylpropanoid metabolism. Besides invert sugars, alpha-amino nitrogen (N) content is considered as an impurity that lowers the sugar beet root quality [18]. Hence, breeding strategies have focused on reducing the alpha-amino N content, which includes free amino acids and betaine levels in the root [19]. However, this may impact plant performance when subjected to abiotic stress, since free amino acids improve resistance by osmoregulation, as nitrogen reserves, and as secondary metabolite precursors [20].

The mechanisms underlying the influence of the variability among sugar beet varieties in their storage capacity have been so far related to cell wall stability and cell anatomy, pathogen susceptibility, and carbohydrate metabolism. The latter involves differences in enzyme activities and respiration rate [15, 21]. However, their impact on the metabolic patterns displayed by beet roots and thus, on their quality, remain to be addressed. In this work, we investigated metabolic patterns of sugar beet varieties with contrasting storage capacities, to provide molecular insights underlying an improved beet root storability. For this purpose, six sugar beet varieties were selected displaying well, moderate, or bad storability, as defined by their sucrose loss during storage (cf. Madritsch et al. (2020) [17]). We analyzed primary and secondary metabolites in the selected varieties immediately after harvest, as well as the changes occurring during storage. These metabolite patterns included free amino acids, organic acids, and semi-polar metabolites. Thus, we identified metabolites that may promote an improved storage capacity in sugar beet roots. In addition, we integrated metabolic and transcriptomic data [17] and pinpointed molecular mechanisms that may underlie the storage-promoting effect of detected metabolites.

## Results

### Free amino acids correlate with higher storage capacity of beet roots at harvest

**Fig. 1:**
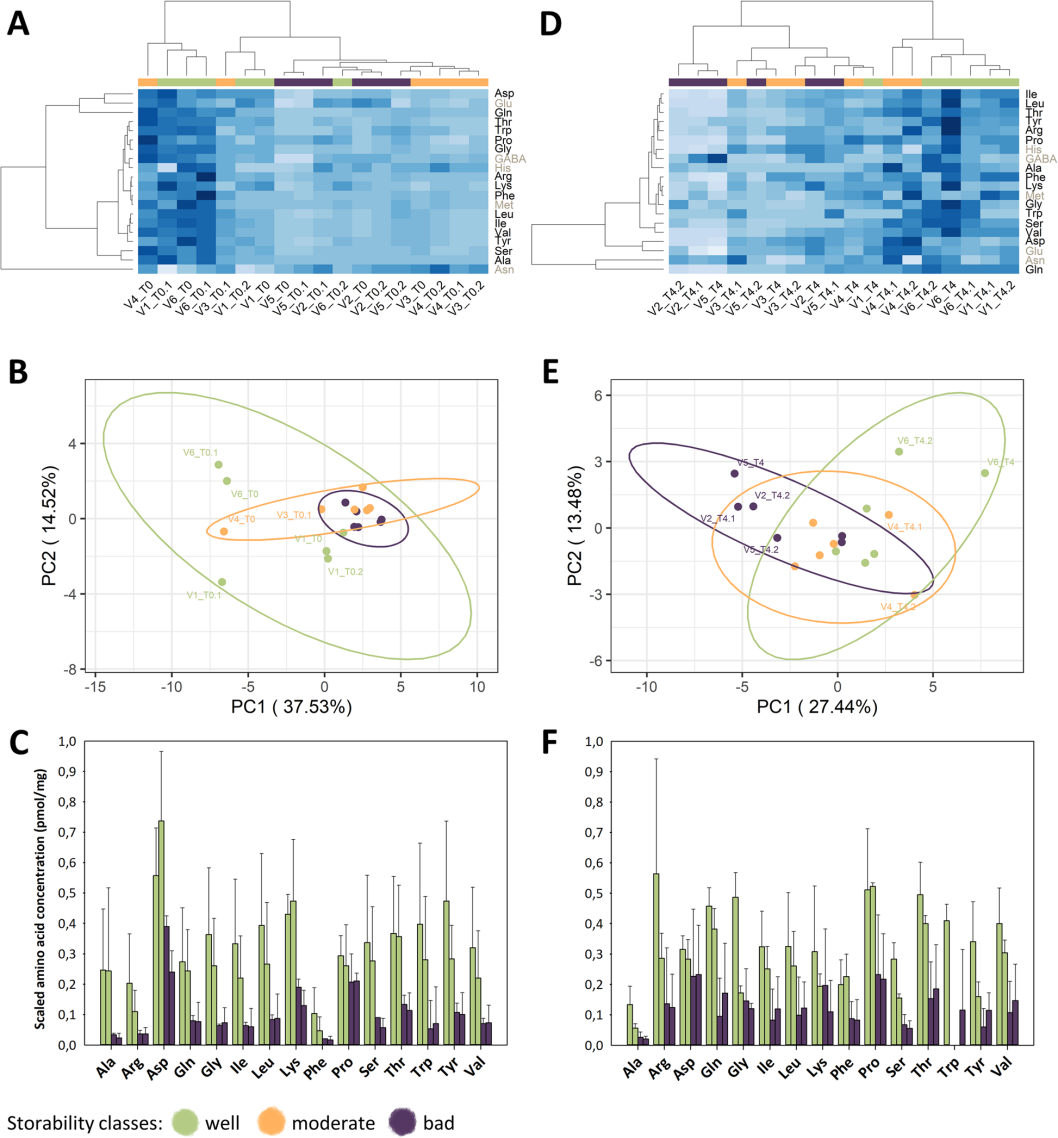
Free amino acid content in sugar beet roots at T0 (left panel, **A**-**C**) and T4 (right panel, **E**-**F**). **A**, **D** Heatmap of free amino acid concentrations clustered by average and labeling seven amino acids that do not correspond to storage capacity in grey. **B**, **E** PCA plot colored by storability. **C**, **F** Scaled free amino acid concentrations of 15 amino acids comparing well and badly storable varieties. Error bars represent standard deviation. From left to right varieties are presented in the following order: V6 (green bar), V1 (green bar), V2 (purple bar), V5 (purple bar)

A total of 22 free amino acids were analyzed in beet roots from well, moderately, and badly storable varieties at five different timepoints (T0 to T4) during storage (Table 1). The results revealed clear differences across the contrasting storage classes already at harvest (T0) (Supplemental Fig. 1). The levels of Aspartate (Asp), Asparagine (Asn), and Glutamine (Gln) were highly abundant in all varieties, followed by Alanine (Ala), Glutamate (Glu), and Serine (Ser). In contrast, Arginine (Arg), Lysine (Lys), Phenylalanine (Phe), and Methionine (Met) were present in lower abundance, whereas Cysteine (Cys) and Norvaline (Nor) were almost absent. At T0, well, moderate, and badly storable varieties formed distinct storage class-specific clusters reflecting their free amino acid levels (Fig. 1A). Overall, well storable varieties showed higher levels of free amino acids. This result was supported by a PCA plot (Fig. 1B), in which the well storable varieties clustered to the left side and the badly storable varieties to the right along the first component (PC1). This accounted for 37.53% of the total variance. Among the well storage varieties, V1 clustered to the lower and V6 to the upper side of PC2, while V2 and V5 did not spread along PC2, which accounted for 14.52% of the total variance. A total of 15 out of the 22 analyzed amino acids were more abundant in the well storable varieties (Fig. 1C): Ala, Arg, Asp, Gln, Glycine (Gly), Isoleucine (Ile), Leucine (Leu), Lys, Phe, Proline (Pro), Ser, Threonine (Thr), Tryptophan (Trp), Tyrosine (Tyr), and Valine (Val). Other free amino acids, Asn, Cys, Gamma-Aminobutyric acid (GABA), Glu, Histidine (His), Met, and Nor, did not show this trend. The moderately storable sugar beet varieties displayed intermediate amino acid concentrations between those shown by well and badly storable varieties (Supplemental Fig. 1). Owing to the high variability inherent to field samples, we analyzed ten additional replicates per variety at T0. These results confirmed that well storable varieties displayed significantly higher amino acid levels already at harvest when compared to varieties with lower storability (Supplemental Fig. 2, Supplemental Tables 1 and 2). Since 15 out of 22 free amino acids showed variety-specific patterns at T0, we analyzed the amino acid profiles after storage to understand the relation of these initial differences in beet root storability.

**Table 1:**
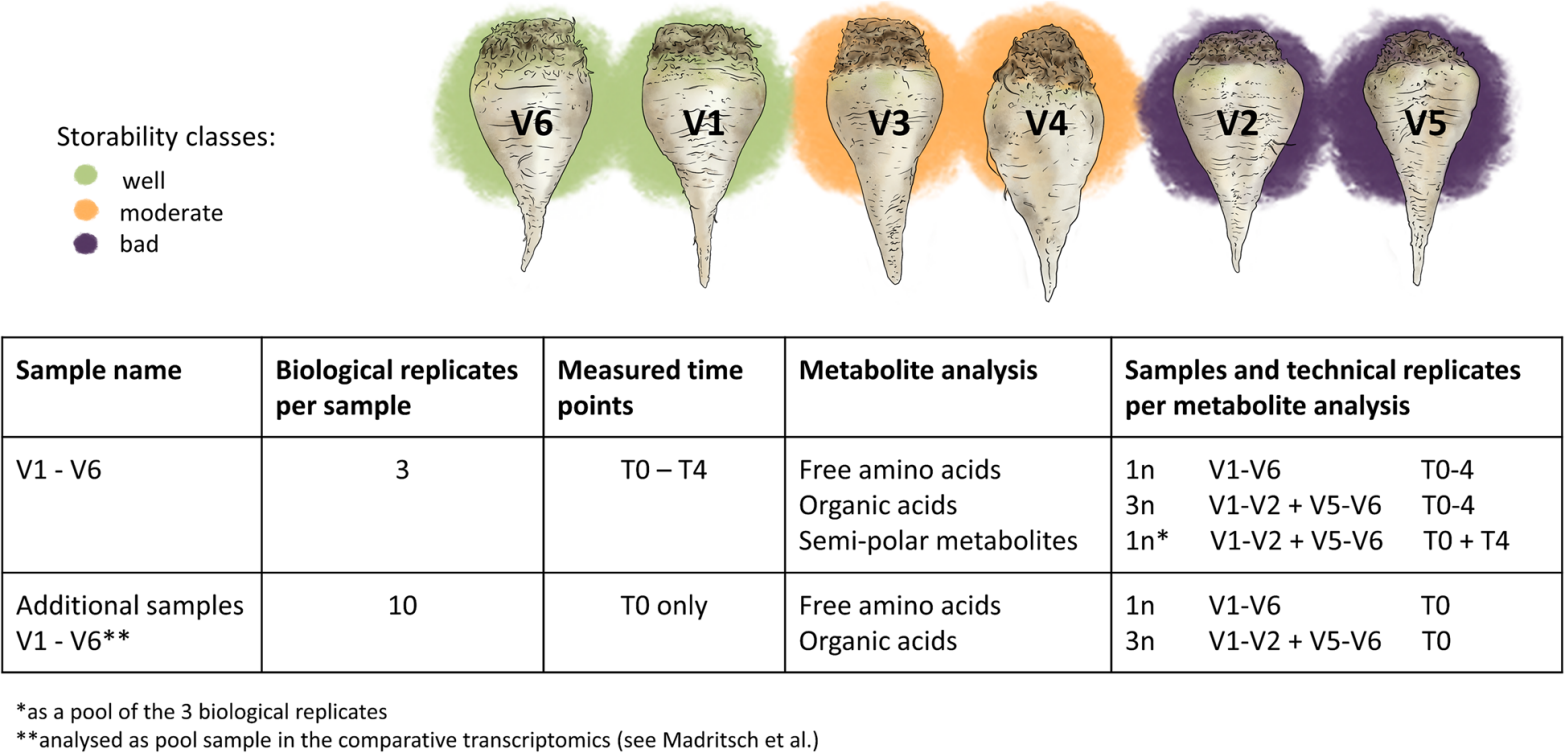
Description of the analyses performed in this study. Beet roots from six varieties (V1-V6) of three different storability classes were analyzed (green: well, orange: moderate, purple: bad)

**Fig. 2:**
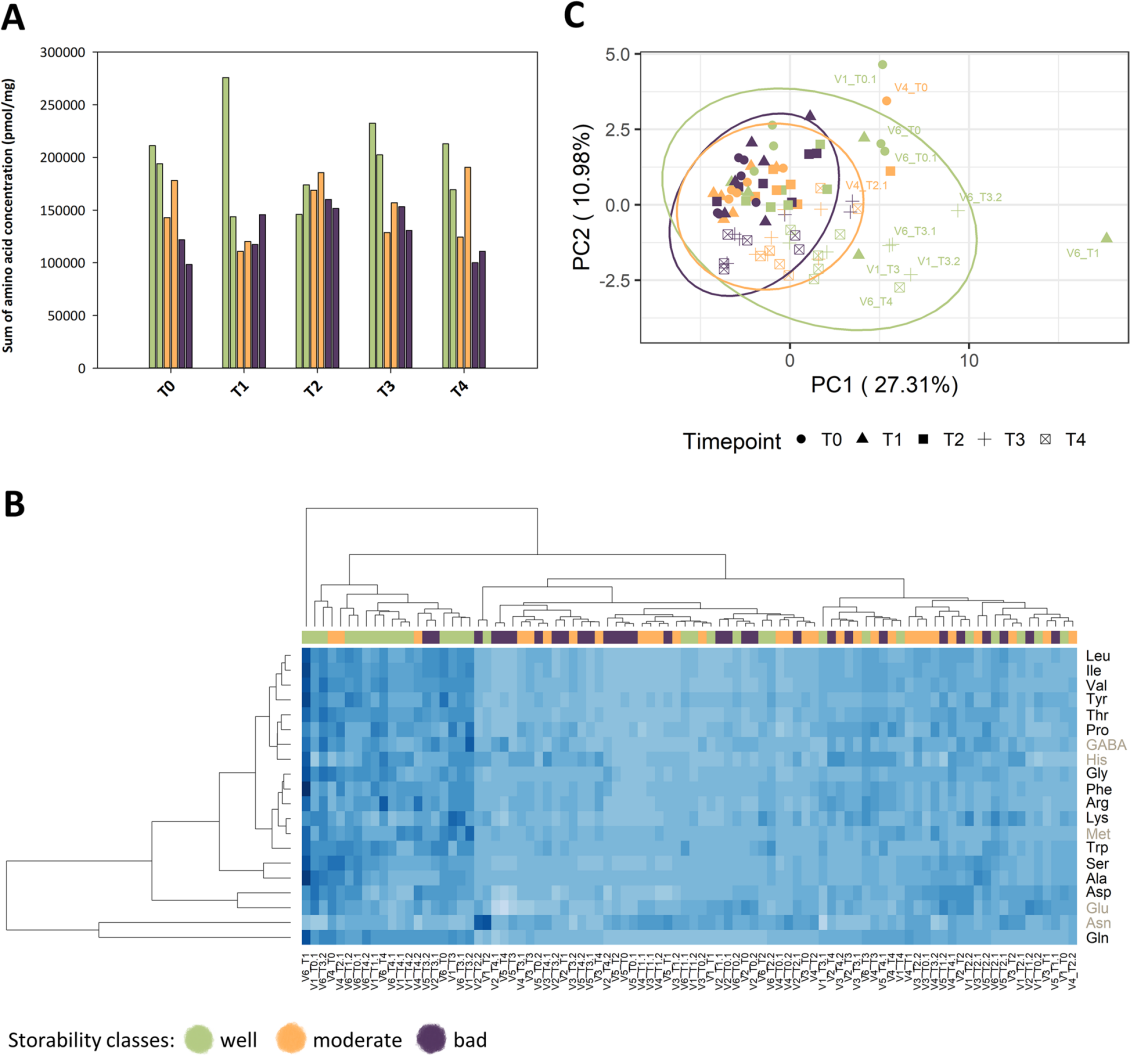
Free amino acid content in sugar beet roots at T0-T4 comparing well, moderate, and bad varieties. **A** Summary of 22 free amino acids. Free amino acid concentrations of each variety were summarized at each timepoint. From left to right varieties are presented in the following order: V6 (green bar), V1 (green bar), V2 (purple bar), V5 (purple bar). **B** PCA plot colored by storability. **C** Heatmap is clustered by average labeling seven amino acids that do not correspond to storage capacity in grey

### Free amino acids maintain similar patterns before and after storage

Initial differences in free amino acid content was still visible after the end of the recorded storage period, but to a weaker extent. When displayed in a heatmap, the initial separation between well and badly storable varieties was not as obvious as at T0 (Fig. 1D). Well storable varieties still formed a separate cluster, but the moderately and badly storable ones showed a more intermixed pattern. This was supported by a PCA plot where more outliers of the initial trend could be seen (Fig. 1E). While PC1 accounted for 13.48% of variance at T0, it reduced to 27.44% at T4. Free amino acid concentrations were rather similar at the beginning (T0, at harvest) and at the end of the storage trial (T4, after 13 weeks of storage) (Fig. 1C and F). Compared to the bad storable varieties, the 15 amino acids that were higher in well storable varieties at T0, were still more abundant at T4 (Fig. 1F and Supplemental Fig. 3).

**Fig. 3:**
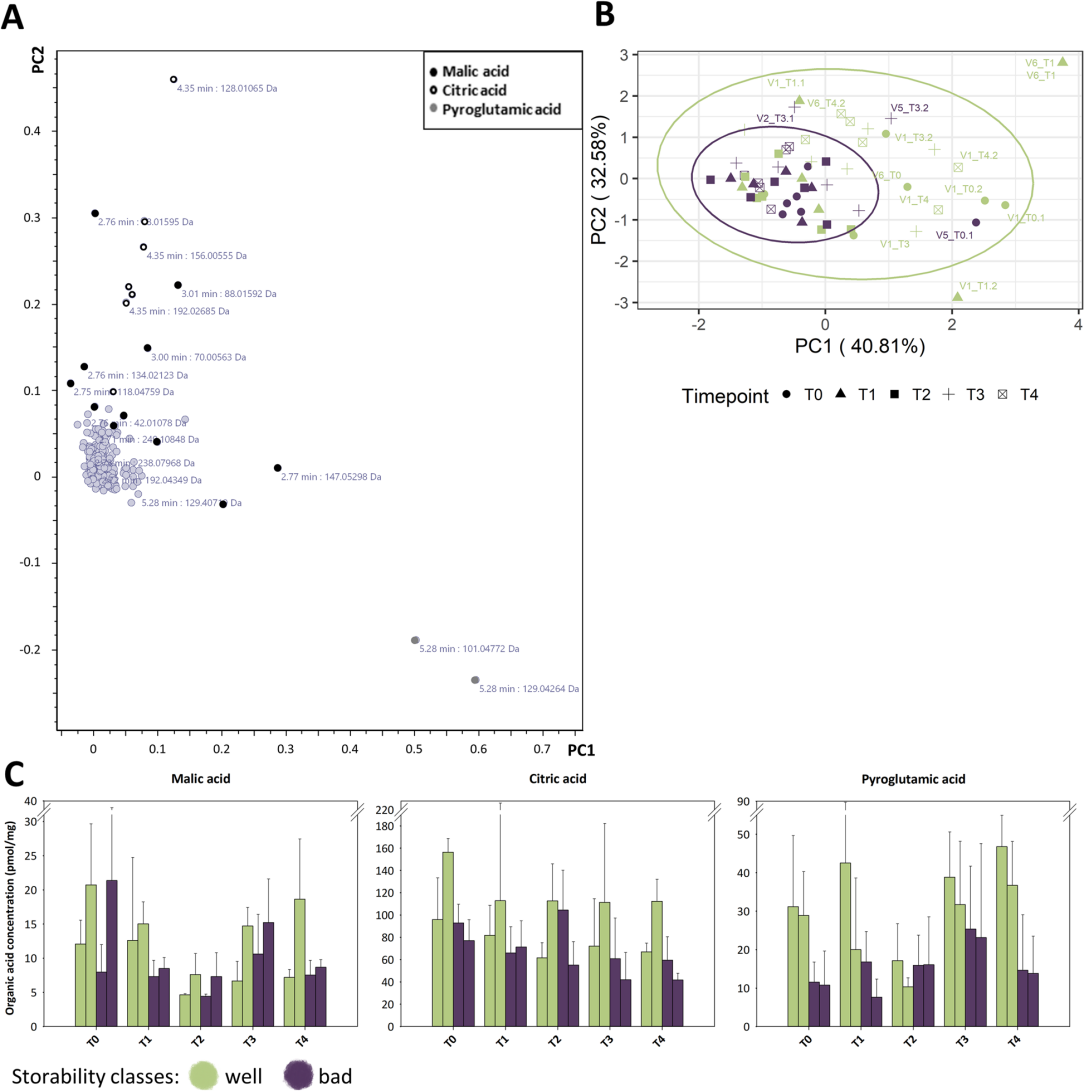
Organic acid content in sugar beet roots comparing well and badly storable varieties at T0-T4. **A** Loadings plot with each point corresponding to detected masses and their retention times. All masses from 0-2.6 min refer to method background and were excluded. **B** PCA plot of three organic acids colored by storability and shaped by storage time. **C** Organic acid concentrations of three organic acids. Error bars represent standard deviation. Varieties are presented from left to right in the following order: V6 (green bar), V1 (green bar), V2 (purple bar), V5 (purple bar)

During storage, the beet roots from different storability classes showed minor variations in their total amino acid levels (Fig. 2A). Nonetheless, the well storable varieties consistently showed higher amino acid levels when compared to varieties with a bad storability. When visualizing the contribution of the different amino acids in a heatmap, the most dominant cluster was still formed by individuals from the well storable varieties at different timepoints (Fig. 2B). Furthermore, two distinct clusters corresponding to the early and late storage phases were recognized, differentiating T0 and T2 from T3 and T4. This was supported by the respective PCA plot of all free amino acid results (Fig. 2C). In this plot, a few storage-dependent changes were observed by the timepoints T3 and T4 which clustered at the lower, and T0-T2 which clustered at the upper side of PC2. The different loading plots that were prepared based on the PCA plot results also indicated minor individual fluctuations of the free amino acids during the storage duration (Supplemental Fig. 4). In view of the clear tendencies detected for free amino acids, further measurements were conducted to ascertain if primary or secondary metabolites also exhibit variety-specific patterns during storage.

**Fig. 4:**
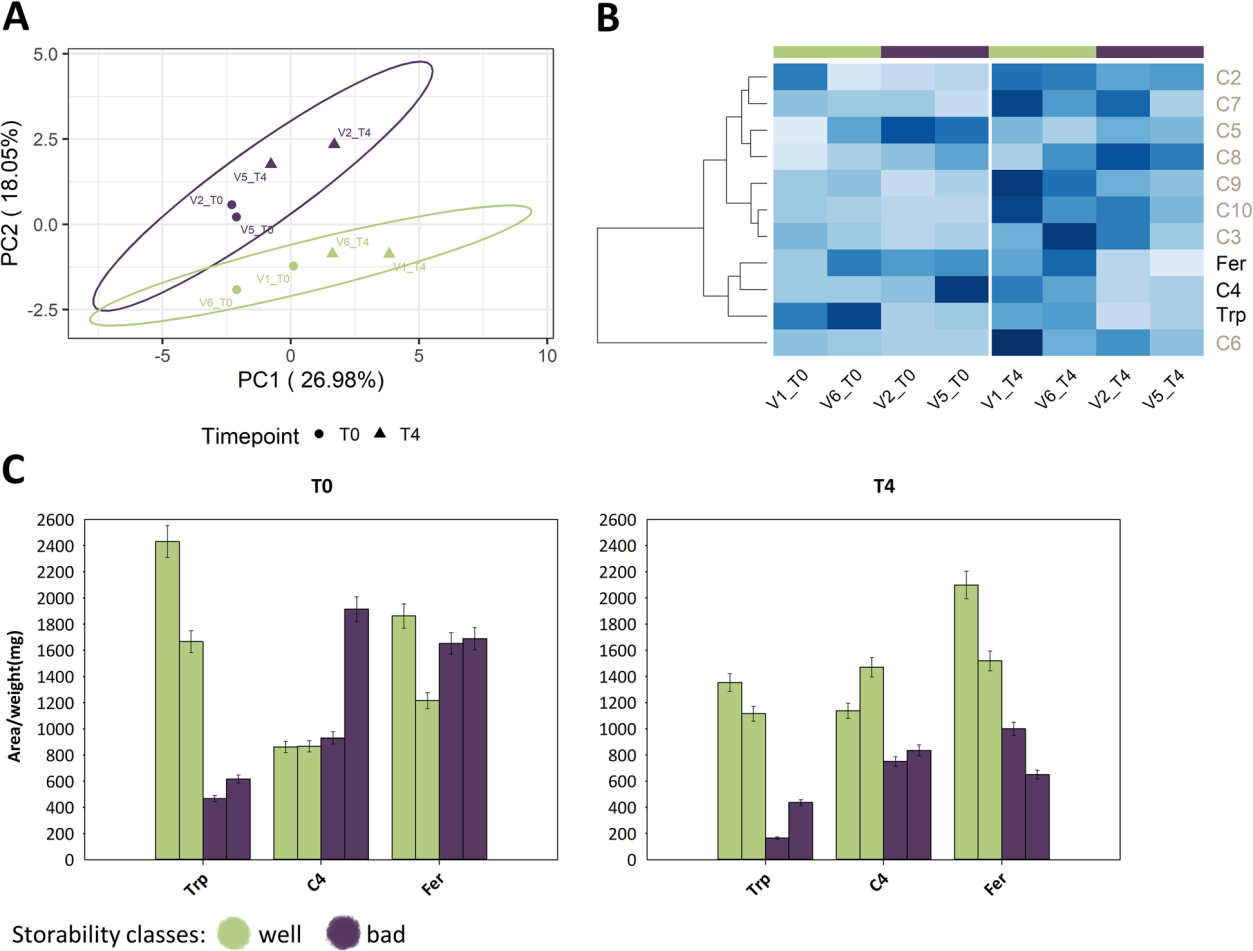
Area per dry weight of semi-polar compounds detected at 280 nm in sugar beet roots comparing well and badly storable varieties at T0 and T4. **A** PCA plot colored by storability. **B** Heatmap clustered by average and grey-marked compounds do not behave in a variety-specific manner. **C** Area per dry weight of three semi-polar compounds that behave in a variety-specific manner. Error bars represent 5%-error since biological replicates were pooled. From left to right varieties are presented in the following order: V6 (green bar), V1 (green bar), V2 (purple bar), V5 (purple bar)

### Untargeted analysis of organic acids and semi-polar metabolites reveal storability-related metabolites

Organic acids and semi-polar metabolites were much less abundant in comparison to free amino acids and especially to sugar content. Therefore, enhanced time- and resource-consuming pre-treatments of the samples as well as an enhanced method development were needed (Supplemental Fig. 5). Nonetheless, metabolite patterns were detected that differ in a storability class-specific manner and that change during storage.

**Fig. 5:**
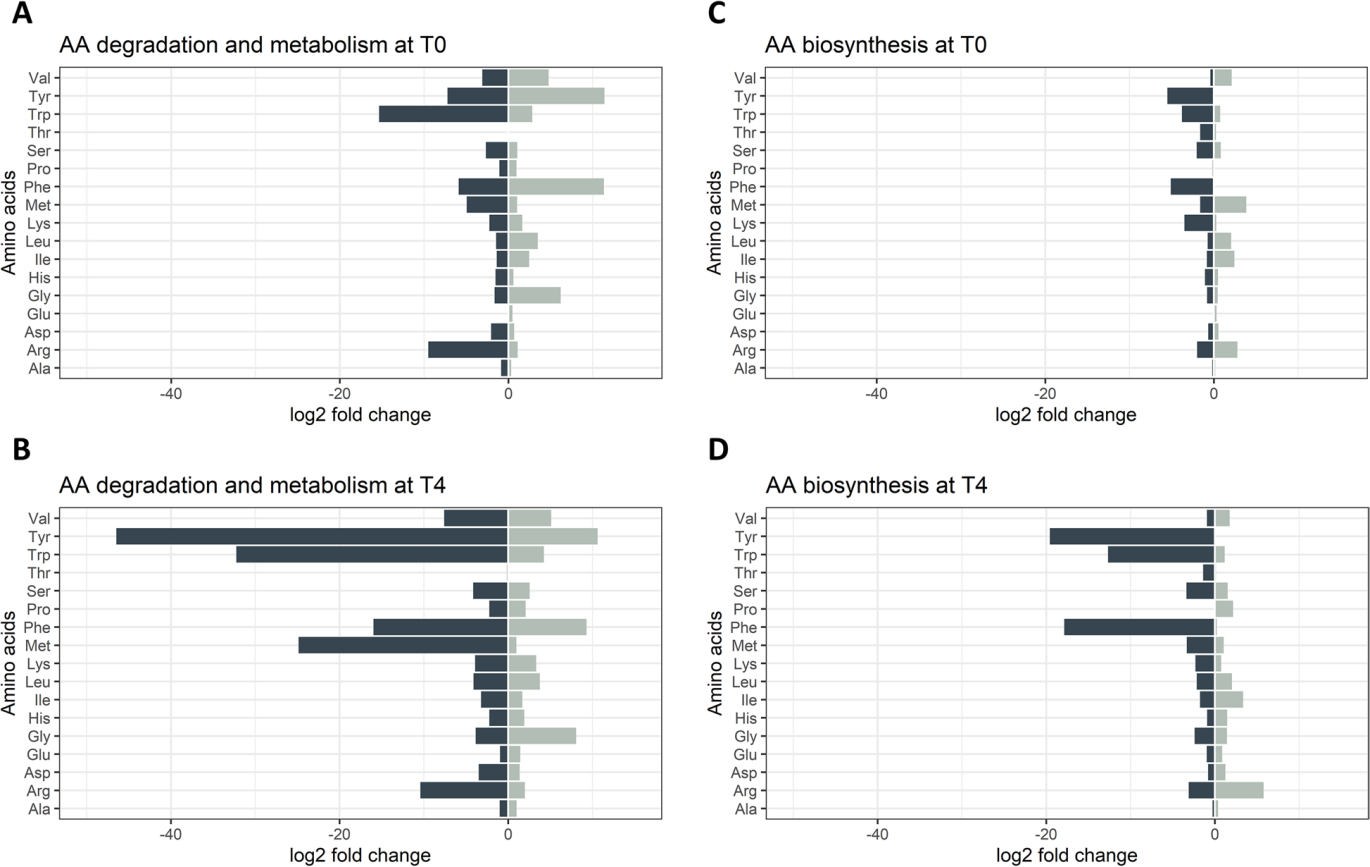
Gene expression patterns of amino acid biosynthesis, degradation and metabolism pathways for the 15 selected free amino acids. Log2 fold change values of well storable varieties compared to badly storable varieties are shown for the amino acid biosynthesis pathway at T0 (**A**) and at T4 (**B**), and for the amino acid degradation & metabolism pathway at T0 (**C**) and at T4 (**D**). Positive log2 fold change values (representative for an upregulation of genes) are visualized in grey, negative values (representative for a downregulation of genes) in black

Owing to the close relation between primary metabolism and cell respiration, and thus, the influence it may have in beet root storability, we analyzed the organic acid composition of the different varieties during storage. An untargeted metabolic profile of polar beet root extracts that were cleaned and enriched in organic acids via solid-phase extraction (SPE) cartridges was obtained. A total of eight PDA peaks at 210 nm and 5,261 metabolic features were identified, amongst which most of them corresponded to ions derived from three metabolites (Fig. 3A). Using commercial standards these were confirmed as citric, malic and pyroglutamic acids. A PCA plot of these three organic acids revealed that their abundance differed between well and badly storable varieties (Fig. 3B). During the storage period, organic acid patterns fluctuated, which can be seen in Fig. 3, C: Citric acid levels constantly declined from T0 to T4, while malic and pyroglutamic acids declined from T0 to T2, but increased from T2 to T4. However, no storage class-specific differences were observed for malic and citric acid. In contrast, pyroglutamic acid differed in a variety-specific manner, exhibiting higher concentrations in well storable varieties at T0 and T4 (Supplemental Fig. 6, Supplemental Tables 3 and 4). This trend is similar to the higher concentrations of 15 amino acids that were mentioned before, i.e. glutamine (Supplemental Fig. 7). When visualized via heat map, storability class-specific clusters appeared for CA and PGA (Supplemental Fig. 8).

**Fig. 6:**
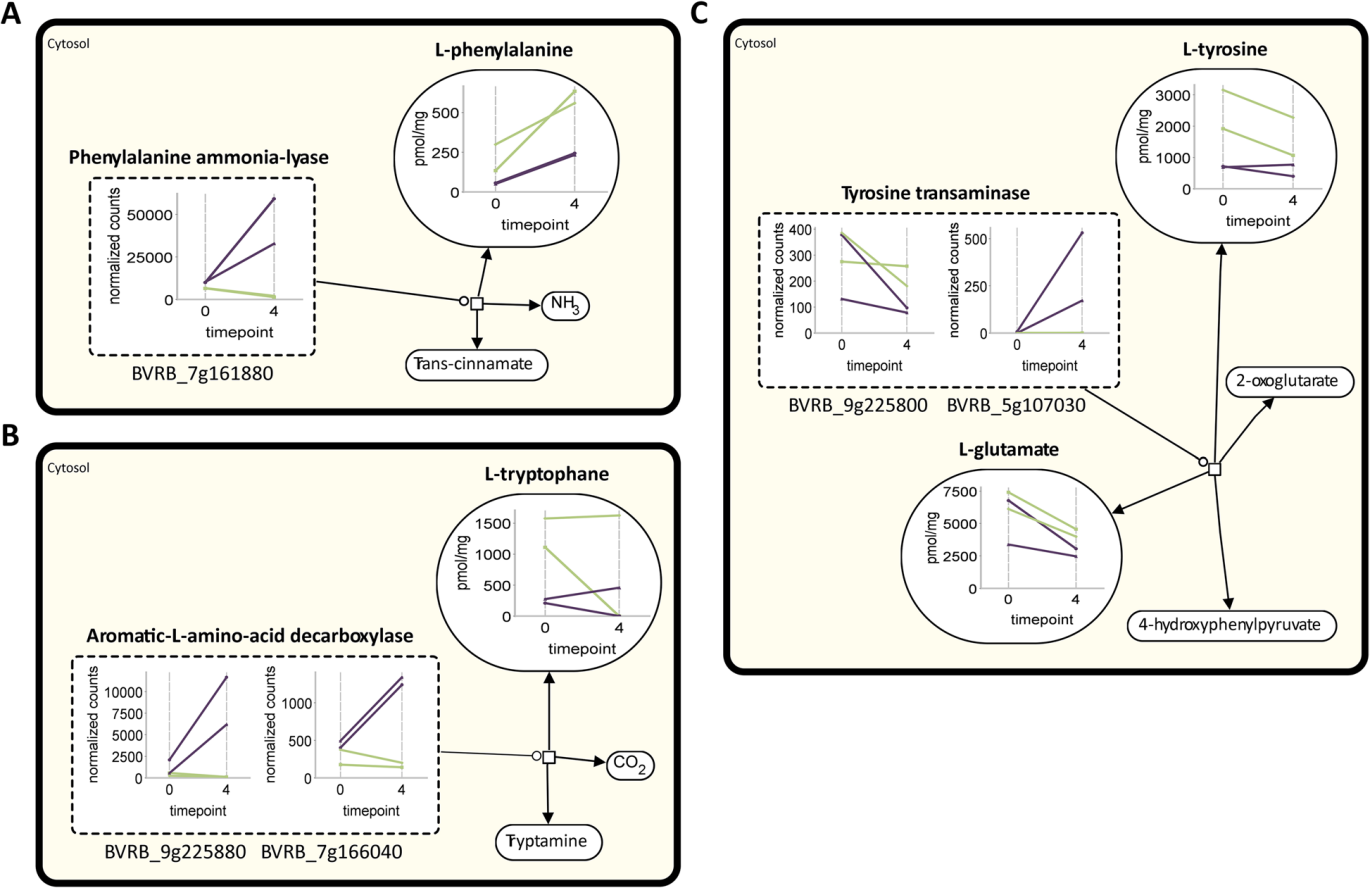
Integrative visualization of selected transcripts (in squares, dashed line) and metabolites (in circles) of part of the degradation pathways of the aromatic amino acids Phe (**A**), Trp (**B**), and Tyr (**C**) using the software tool VANTED. Comparison was done between T0 and T4 (x-axis) and between the well (green) and badly (purple) storable varieties

Besides the free amino acids and organic acids, a variety of secondary metabolites were also analyzed. This includes those compounds which are commonly involved in both biotic and abiotic stress-induced plant defense. Madritsch et al. (2020) reported significant changes during storage in the categories of lignin and phenylpropanoid metabolism [17]. Therefore, we performed an untargeted LC–MS analysis to investigate if secondary metabolites like phenylpropanoids may influence the sugar beet storage capacity. We analyzed semi-polar plant extracts which contained different primary and secondary metabolites, including amino acids and several phenolic compounds. To get a better understanding regarding their possible involvement in the storage capacity of sugar beet, they were analyzed in pooled samples (composed of three biological replicates) of well and badly storable varieties at T0 and T4. Their analysis revealed 9,454 mass features as well as 12 PDA peaks at 280 nm.

A PCA plot of the 12 UV-absorbing semi-polar metabolites indicated a separation based on storage time and storability class (Fig. 4A). The well storable varieties clustered along the lower side of PC2, while the badly storable varieties were located on the upper side. Furthermore, samples analyzed at T0 clustered to the left and at T4 to the right side along PC1. This is supported by a heatmap (Fig. 4B), showing that storage time-specific differences seemed to be mostly influenced by seven out of 12 detected compounds, which were increased at T4 (Supplemental Fig. 9). Storability class-specific differences could be mainly dedicated to three semi-polar metabolites: Trp, C4, and ferulic acid (Fer) (Fig. 4C). As described before, Trp had high levels in well storable varieties at T1 and T4. C4 and Fer were also elevated in well storable varieties, but only at T4 (Fig. 4C).

Based on the combined free amino acid, organic acid and semi-polar metabolite results, patterns were identified that changed during storage and that differed in a storability class-specific way. Subsequently, a detailed analysis of the molecular pathways underlying metabolite changes was pursued by integrating metabolite and transcript information.

### Higher amino acid levels in well storable varieties are likely due to downregulated degradation and metabolism pathways

The analysis of the corresponding transcript data revealed an increase in the levels of genes involved in amino acid degradation and downstream metabolism pathways (Fig. 5A and B) with both, up- and downregulated genes which was in contrast to the genes involved in amino acid biosynthesis (Fig. 5C and D). Regarding the latter, the well storable varieties showed an increased downregulation of the corresponding genes at both timepoints, T0 and T4. At T0 (Fig. 5C), Tyr and Phe exhibited the most reduced levels in the expression of biosynthetic genes, with log2 fold change values (log2fc) of -5.5 and -5.09, respectively. This trend intensified towards T4 (Fig. 5D), where a stronger downregulation of the gene expression of well storable varieties (which means a higher upregulation in the badly storable varieties) was observed, especially for genes involved in Tyr, Trp, and Phe biosynthesis, with log2fc values of -19.56, -12.67, and -17,85, respectively. In contrast, an upregulation of certain biosynthetic genes was observed for Val, Leu, Ile, and Arg at T0 (Fig. 5C), and especially for Arg at T4 (Fig. 5D) of the well storable varieties in comparison to the badly storable ones.

The gene expression pattern of the amino acid degradation and amino acid metabolism pathways followed a similar trend to those described in the previous paragraph. At T0 (Fig. 5A), an increase in the downregulation in the well storable varieties was observed for Trp (log2fc: -15.33) and Arg (log2fc: -9.49). In addition to the observed downregulation, the Tyr, Phe and Gly degradation and metabolism pathways showed a larger amount of active (upregulated) genes in the well storable varieties at T0 (log2fc values: Tyr: 11.39 | -7.21, Phe: 11.32 | -5.9, Gly: 6.21 | -1.63). The differences between well and badly storable varieties were present at T4 (Fig. 5B), where a prominent downregulation of genes belonging to the Tyr, Trp, Phe, and Met degradation and metabolism pathway occurred for the well storable varieties, with log2fc values of -46.46, -32.22, -15.96, and -24.83.

The combinatorial visualization of selected transcriptomic and metabolomic data (Fig. 6) supports the findings described above. The degradation of the aromatic amino acids Phe, Trp and Tyr, are altered in parallel with the storage-capacity related expression of a Phe ammonia-lyase (Fig. 6A). A similar storage-capacity related pattern was observed for genes annotated as aromatic-L-amino-acid decarboxylases, revealing an upregulation in badly storable varieties especially at T4 (Fig. 6B). Genes annotated as tyrosine transaminase were expressed similarly in the Tyr degradation pathway (Fig. 6C).

### Protein degradation processes are suppressed in well storable varieties

From the 324 *Beta vulgaris* genes falling under the GO category “proteolysis” (GO:0006508), 35 genes were significantly differentially expressed between well and badly storable varieties (Supplemental Table 5). At T0, 22 significantly differentially expressed genes (DEGs) were detected, and at T4, 30 DEGs, with 17 genes showing differential expression at both timepoints. Overall, proteolysis seemed to be suppressed in the well storable varieties, since out of the 22 genes at T0, 15 were downregulated, and out of the 30 genes at T4, 24 were downregulated. Among the latter, two genes were annotated as metalloproteases (BVRB_5g104400: 1-MMP [22], and BVRB_3g050270: OMA1, members of the plant mitochondrial proteolytic machinery [23]), and four genes could be assigned to basic 7S globulin (BVRB_5g100060, BVRB_5g108660, BVRB_9g211890, and BVRB_008150), which is described to be expressed upon wounding and heat [24]. Only one gene showed a significant downregulation at both timepoints in the well storable varieties: BVRB_6g149220, which was identified as an aspartic proteinase CDR1-like gene. In *A. thaliana*, CDR1 is known to be induced in response to pathogen infestation [25]. Besides the many downregulated genes, one gene was significantly upregulated in the well storable varieties at T0 (BVRB_8g181870), annotated as serine carboxypeptidase-like 7, that has been described to be expressed during leaf senescence [26], and one at T4 (BVRB_1g020870), annotated as CO_2_-response secreted protease, which is described to cleave the pro-peptide EPF2 upon elevated CO_2_, in turn repressing stomatal development [27].

## Discussion

Pools of free amino acids are tightly controlled through relevant pathways including amino acid and protein biosynthesis as well as degradation. The direction of amino acid metabolism and the size of free amino acid pools highly depends on the type of abiotic stress [20, 28]. Combining transcriptome and metabolome data indicates which pathways are induced or repressed during abiotic stress and the source of free amino acid pools [28]. While induced pathways may contribute to stress protection, repressed pathways and degraded molecules are rather irrelevant.

### Higher free amino acid levels likely confer better storability

Our results of free amino acid analysis support a positive correlation between the sugar beet storage capacity and the size of its amino acid pools at different time points. There are indications that larger pools of 15 free amino acids could be associated with a good storage capacity. Alternatively, smaller pools are attributed to bad storage capacity. Moreover, contrasting amino acid levels can be detected already prior to storage at T0. This suggests that the storage capacity is already determined before storage. There may be two explanations for higher concentrations of free amino acids in well storable varieties prior to storage: either this is triggered by external factors during cultivation, or it is a genetic characteristic. Several environmental factors related to drought, osmotic stress, or fertilization, are known to enhance the amino acid content during sugar beet cultivation [29]. However, these factors can be ruled out here, since all varieties were grown randomly at the same location and were exposed to the same environmental factors. Furthermore, the storage capacity of the varieties has been well documented through storage trials performed by AGRANA Research & Innovation Center (ARIC) over several years. This suggests that the higher amino acid accumulation of well storable varieties could be a genetic characteristic. This is further indicated by the reduced expression of genes belonging to the GO category “alpha-amino acid catabolic process”, especially 13 weeks after storage [17], together with the combinatorial visualization (Fig. 6a-c), suggesting a suppression of amino acid catabolism in well storable varieties. Another possibility for the increased amino acid levels in well storable varieties could be the differential uptake of free amino acids from soil during the cultivation [30], however, this remains to be elucidated. A higher number of parenchyma cells and a smaller cell area were found in well storable varieties (see Table S3 in Madritsch et al. (2022) [17]). In sugar beet roots, the diameter of parenchyma cells correlates with sucrose concentration and water potential [31]. Small parenchyma cells have higher sucrose levels [32]. Furthermore, the water potential of parenchyma cells is regulated via betaine accumulation in the cytosol to balance high sugar concentration in the vacuole [32]. It is likely that, the increased accumulation of free amino acids in the numerous parenchyma cells of well storable varieties could have an osmolarity regulatory effect similar to betaine.

In general, amino acids are either incorporated into proteins or available in the free form in a plant cell. Free amino acids could contribute to abiotic stress resistance as precursors for protein synthesis, signaling and specialized metabolites supporting growth and defense [33]. Furthermore, they promote osmoregulation and act as nitrogen or carbon reserves. Among them, proline levels have been observed to increase due to osmotic stress in sugar beet [34–36]. In beet roots, increased proline levels could indicate drought stress and an excess nitrogen supply [37]. Elevated proline levels occur in many plant species during abiotic stress and in some of them, proline is expected to play a key role in active stress adjustment [35]. In the present study, proline was more abundant in the well and lower in the badly storable varieties before storage (at T0) and this difference was even more pronounced following 13 weeks of storage (at T4). This indicates a better stress protection in well storable varieties through proline acting as osmolyte [38].

We found that the most abundant free amino acids in sugar beet roots were Ala, Asp, Gln, Ser, Glu and Asn, supporting results published in previous studies [39]. Among them, Ala, Asp, Gln and Ser were more abundant in well and lower in badly storable varieties before and after storage (at T0 and T4 respectively). Glutamine is abundant in many plant species [40]. It is synthesized from glutamate and ammonia contributing to the biosynthesis of proteins, nucleic acids, amino acids, and other nitrogen-containing compounds [19]. In sugar beet roots, glutamine is a main component of the alpha-amino N content. Genotypic differences in soluble nitrogen can be observed as early as 100 days after sowing and correlates with glutamine synthetase activity [18, 19]. In the study by Madritsch et al. (2022) the alpha-amino N content behaved similarly to free amino acid levels including glutamine [17]. Glutamine is a major donor and may contribute to a high quality storage capacity by supporting amino acid production. However, we did not find a correlation between elevated alpha-amino N content and glutamine synthetase activity based on transcriptomic data.

### Badly storable varieties are characterized by a higher aromatic amino acid turnover

In addition to proline and glutamine, the aromatic amino acids Trp, Tyr, and Phe were highly abundant in well storable varieties before and after storage (at T0 and T4). Aromatic amino acids derive from the shikimate pathway [41]. Besides protein biosynthesis, they are precursors of pigments, alkaloids, phenylpropanoids, hormones, and cell wall components like lignin [42]. Related gene expression patterns point towards an elevated turnover of these amino acids in badly storable varieties through an upregulation of biosynthesis, consumption, and a stabilization of the amino acid pools in the well storable varieties through a downregulation of genes belonging to the GO category “aromatic amino acid family metabolic process” (cf. Figure 5 C in Madritsch et al. (2020) [17]). That badly storable varieties show a higher metabolism rate can be supported by the comparative transcriptomics study, where a higher energy production and consumption in badly storable varieties during storage is proposed [17]. It is likely that the free amino acid pools of the badly storable varieties are mainly affected by an elevated proteolytic activity, which was observed in the transcriptomic data of the present study. It is well known that proteolysis is increased in response to stress, especially if carbohydrates are scarce [43]. Since two members of the plant mitochondrial proteolytic machinery were significantly downregulated in the well storable varieties, an elevated use of amino acids as alternative substrates for mitochondrial respiration in the badly storable ones is likely.

In general, aromatic amino acids and related specialized metabolites increase due to abiotic stress, and they may contribute to abiotic stress tolerance in plants [44–46]. This suggests a contribution of Trp, Tyr, and Phe to a good storage capacity by promoting secondary metabolite biosynthesis. So far, not much is known about the role of aromatic amino acids and their secondary metabolites in sugar beet roots during storage. In the related study by Madritsch et al. 2020, lignin content was found to be higher in the badly and lower in the well storable varieties at T0 [17]. During storage, lignin concentrations decreased in all varieties, but to a stronger extent in the badly storable ones. Lignin is a product of Phe and a major cell wall component that confers biotic and abiotic stress protection. Furthermore, PAL expression was significantly elevated in the badly storable varieties during storage. Based on this combination of observations it is likely that higher levels of Phe in well storable varieties contribute to lignin biosynthesis. This is further supported by a KEGG enrichment analysis, which revealed a downregulation of genes related to secondary metabolites including all stages of the phenylpropanoid pathway as well as flavonoid biosynthesis in well storable varieties [17]. This suggests that high levels of aromatic amino acids are not mainly directed into phenylpropanoid or flavonoid metabolism after 13 weeks of storage (at T4). However, according to the GO enrichment analysis in Madritsch et al. (2022), other secondary metabolic processes are upregulated in well storable varieties at T4 [17]. These include terpene metabolic process, carotene biosynthetic process, steroid metabolic process, and isoprenoids secondary metabolites. The enhanced pools of aromatic acids may indirectly feed into these processes, e.g. via their degradation to fumarate. Indeed, the transcript levels encoding four enzymes for the degradation of Phe, Trp, and Tyr is higher in the badly storable varieties. Gene expression of secondary metabolism related transcripts may be regulated at the post-transcriptional level [41], which would elude the transcript analysis performed herein. One indication for this possibility is the higher concentration of C4 and Fer after 13 weeks of storage in the well storable varieties.

Like free amino acids, α-amino N content is higher in the well and lower in the badly storable varieties before and after storage (at T0 and T4) and it remains stable during storage [17]. The alpha-amino N content is a summary of soluble amino acids and amides and its accumulation during sugar beet storage is affected by temperature, storage duration, and genetics (the sugar beet variety itself) [7, 18, 47, 48]. High storage temperature (approximately 20 °C) and elongated storage (56–110 days) increase the alpha-amino N content due to protein and sucrose degradation. Since the beet roots in this study were stored at low temperature (5–13 °C), a low accumulation of the alpha-amino N was expected. In addition, enhanced catabolism of free amino acids could reduce alpha-amino N concentrations and explain the patterns observed in the present study [48].

### Pyroglutamic acid and two semi-polar metabolites contribute to well storage capacity

In addition to the free amino acids, organic acids and semi-polar metabolites were also investigated in this study. Organic acids are a dynamic class of primary metabolites that contribute to the energy status and the redox balance of plant cells. They are products of the citrate cycle, which provides substrates for amino acid synthesis linking organic and amino acid metabolism. We found that citric, malic and pyroglutamic acid were the three most abundant organic acids in sugar beet roots, similar to previous reports [49]. Pyroglutamic acid exhibited higher levels for well and lower for badly storable varieties at T0 and T4, suggesting a contribution to a good storage capacity in sugar beet. Pyroglutamic acid is formed from activated glutamate or by the degradation of glutathione and it serves as a precursor for both [50]. It is regulated in plants as a response to biotic and abiotic stress [51–53]. However, the biological function of pyroglutamic acid within sugar beet metabolism is difficult to define since it can also derive from spontaneous chemical conversions of Gln and Glu [54]. This is indicated by very similar Gln and pyroglutamic acid patterns in the analyzed samples.

Certain compounds within the semi-polar metabolites (C2, C3, and C6-10) were increased in both, well and badly storable varieties after 13 weeks of storage (at T4). Consequently, they may contribute to the storage capacity, but not in a storage class-specific manner. Besides Trp, two compounds, C4 and Fer, were discovered to have increased levels for well storable varieties after 13 weeks of storage. There are indications that phenylpropanoids influence biotic and abiotic stress resistance in sugar beet, e.g. against *Cercospora beticola* or during salt stress [55, 56]. Hence, an involvement during sugar beet storage may be possible. Ferulic acid derives from the phenylpropanoid metabolism in plants, it has antioxidant properties, and it can be incorporated into lignin [57–60] . Furthermore, it builds crosslinks between carbohydrates and lignin in plant cell walls, which confers stability [61]. In sugar beet, differences in the composition of cell wall bound phenolics, including ferulic acid, were already shown, but they did not correlate with mechanical properties [62]. Still, it can be hypothesized that enhanced ferulic acid content in well storable varieties may improve the cell wall stability, and thereby, raise the storage capacity. Enhanced ferulic acid and C4 levels in well storable varieties have been found at T4 and not at T0 suggesting that this trait was established during storage.

Organic acids and the semi-polar metabolites are rather difficult to analyze in sugar beet roots due to the high sucrose background and their low abundance. This may be one of the reasons why their influence on sugar beet storage capacity has not yet been described in detail. Among the detected organic acids and semi-polar metabolites only pyroglutamic acid, a possible amino acid derivative, and tryptophan, an amino acid, showed a clear storage class-specific trend at T0. This further highlights the role of small amino compounds in the root metabolism during storage.

### Free amino acids are more than just impurities

In general, soluble nitrogen-containing compounds like free amino acids are considered as impurities because they lower sugar beet quality and negatively influence industrial processing [18, 19]. Hence, sugar beet roots containing abundant levels of free amino acids are rather un-suited for sucrose extraction. However, the harvested root needs certain reserves to survive the storage period and our results indicate that free amino acids are a major part of them. Furthermore, if free amino acids positively influence the sucrose content during the storage period, they also support the beet root quality to a certain extent. Since breeding just considers low alpha-amino N, more knowledge is needed about the influence on free amino acid pools and other nitrogen-containing compounds [19]. Our findings encourage to test free amino acid content in a higher number of well and badly storable sugar beet varieties. Moreover, the obtained results should be verified at different growing sites and during several years. It remains to be investigated if pools containing higher concentrations of free amino acids would actually enhance amino N and we suggest considering the contribution of other factors, such as the contribution of amides.

## Conclusions

We analyzed the free amino acids, organic acids, and semi-polar metabolites in the roots of well, moderate, and badly storable sugar beet varieties stored for 13 weeks. Based on this, we described storage class-specific metabolite patterns and their fluctuations at harvest and after storage. Remarkably, we found that 15 free amino acids and pyroglutamic acid were more abundant in well storable varieties already at harvest but also following 13 weeks of storage. We hypothesize that these metabolites contribute to a high quality storage capacity of sugar beet roots, e.g. by serving as reserves or osmolytes. Furthermore, since these variety-specific differences appeared already at harvest, we conclude that they are defined prior to storage, likely as a genetic characteristic. Besides free amino acids, we propose that phenolic compounds may also impact storability, as indicated by C4 and Fer, which were enhanced in well storable varieties following 13 weeks of storage. In addition, we suggest that citric acid and an additional seven semi-polar compounds generally promote root metabolism since they were similarly altered in both well and badly storable varieties during storage. Using combinatorial metabolomic and transcriptomic analyses, we propose specific molecular mechanisms that may underlie the storage-promoting effects of increased free amino acid pools. These include a transcriptional downregulation of amino acid degradation and metabolism-related genes as well as a higher number of downregulated proteolysis genes in well storable varieties before and after 13 weeks of storage.

## Methods

### Reagents

Chemicals were purchased from Waters (DE), Merck KGaA (DE), Carl-Roth GmbH & Co. KG (DE), and Th. Geyer GmbH & Co. KG (DE). Solvents (acetonitrile, methanol, water, trifluoroacetic acid) were LC–MS grade (min. 99,95%, Chemsolute®, Th. Geyer). Formic acid (min. 98%) was purchased from J.T. Baker (VWR). The analytical standard mix for free amino acid analysis contained 22 amino acids of different polarities and masses (Amino Acid Standard, Waters (DE)). For organic acid analysis the standards were either analyzed as a single stock or a mix of four organic acid compounds of different polarities and masses (Organic acid kit, UPLC grade from Merck KGaA (DE) and L-Pyroglutamic acid ≥ 99.0% from Sigma Aldrich (DE)). For the analysis of semi-polar metabolites, the standard was either a single stock of Trp or a mix comprised of eight phenolic compounds of different polarities and masses, acidified with 0.1% formic acid (v/v) [63].

### Plant material

The samples used in this study were described in Madritsch et al. (2020) [17]. Shortly, six sugar beet (*Beta vulgaris* L.) varieties, V1-V6, were selected that differ in their storage capacity, which was defined by relative sucrose loss (normalized to the average weight loss) over storage time. Five varieties (V1-V5) were provided by Strube Research GmbH & Co. KG., Germany and one (V6) derived from AGRANA Research and Innovation Center (ARIC), Austria. The storage capacity of these varieties was grouped into three classes defined as well (V1 & V6), moderately (V3 & V4), and badly storable (V2 & V5) varieties. Selected varieties were sown in spring 2017 in an irrigated site of Austria (Frauenkirchen) operated by the partner ARIC, they grew in a randomized plot design, and were harvested on 16th October 2017. After harvesting, most of the beets were stored under controlled conditions while mimicking outside temperatures. Samples were taken at five timepoints: directly after harvest, and after 1, 2, 8, and 13 weeks of storage (T0-T4) from three biological replicates per variety per timepoint. Additionally, 10 individuals per variety were taken prior to storage (at T0) and used as additional biological replicates for downstream analysis. Sugar beet root material was collected at the thickest part of the beet, a disk was cut out and further sliced into four small rectangular blocks (1 × 1 × 8 cm) using a French fry cutter. The blocks were immediately frozen in liquid nitrogen. One block per beet was used for each transcriptomic and metabolomic analysis and the remaining parts of the beet root were used for sugar and standard analyses by ARIC. For more details, please see Madritsch et al. (2020) [17].

### Metabolomics

#### Sample preparation

One frozen block (1 × 1 × 8 cm) was used for whole metabolite analysis (see Fig. 1 in Madritsch et al. 2020) [17]. Initially, this block was freeze-dried and homogenized to fine powder using an IKA Tube Mill control.

#### Amino acid analysis

The free amino acid content of sugar beet roots was determined in all samples. Therefore, 10 mg of freeze-dried and homogenized material was extracted in 0,4 ml 100% (v/v) methanol for 30 min at 70 °C with 1,400 rpm agitation. Then, 0.5 volumes of CHCl_3_ and 1 volume water were added following centrifugation at 1,350 rpm and 15 min. Afterwards, 0.1 ml of the supernatant was collected, vacuum-dried and re-dissolved in 20 μL of water for 10 min at 30 °C and 1400 rpm agitation. The derivatization of amino acids was conducted according to the AccQ Tag Ultra derivatization method using the AccQ-Tag Ultra Derivatization Kit, Waters (DE). Shortly, 10 µl of the re-dissolved extract were mixed with 70 µl borate buffer and 20 µl of Waters AccQ Fluor Reagent followed by 1 min incubation at rt and 10 min at 55 °C and 1400 rpm agitation. Finally, 1 µl of the derivatized extract was analyzed via reverse phase ultra-performance liquid chromatography-fluorescence detection (RP-UPLC-FLR). Therefore, an AccQ-Tag Ultra RP Column 130 Å 1.7 µm, 2.1 mm 100 mm (Waters, DE) was used applying a flow of 0.7 ml/min at 35 °C and a gradient composed of AccQ-Tag Ultra Eluent A concentrate (1:10 diluted in water) and AccQ-Tag Ultra Eluent B (both UPLC-grade, Waters, DE) as follows: 0-0.5 min 99.9% A and 0.1% B; 5.7 min 98% A and 2% B; 7.5 min 91% A and 9% B; 8.7 min 90.3% A and 9.7% B; 9.2 min 87% A and 13% B; 12 min 86% A and 14% B; 12.2-12.7 min 40% A and 60% B; 13-14 min 99.9% A and 0.1% B. Derivatized amino acids were detected by fluorescence at 266 nm excitation and 473 nm emission wavelength. Identification and absolute quantification of amino acids was done in comparison with corresponding standard dilutions using Empower 3.0 software (Waters, DE) for the evaluation of chromatographic data sets.

#### Organic acid analysis

Organic acids were analyzed in three technical replicates of four sugar beet varieties (V1, V2, V5 and V6) at all timepoints including the 10 biological replicates before storage. The extraction was similar to amino acids with following changes; 50 mg of freeze-dried and homogenized material were extracted in 0.5 ml 100% (v/v) methanol. The complete supernatant was collected, vacuum-dried and re-dissolved in 0.6 ml of water. After extraction, a purification step was applied prior to LC–MS analysis to reduce sugar contamination and to specifically concentrate organic acids. The protocol was varied from Agius et al. (2018) [64]. Chromabond NH_2_ cartridges of 3 ml volume and 500 mg material from Macherey–Nagel were employed using one cartridge for three technical replicates of one sample. NH_2_ cartridges were conditioned with 4 ml of 100% (v/v) acetonitrile followed by equilibration with 4 ml of 40% (v/v) acetonitrile. The extract was adjusted to 40% (v/v) acetonitrile and completely applied on the NH_2_ cartridge material. After two washing steps of 2 ml of 40% (v/v) acetonitrile followed by 1 ml water, organic acids were eluted in 3 ml of 400 mM trifluoroacetic acid. Finally, 0.8 ml of the cleaned extract elution were concentrated ~ 10 × under vacuum.

The cleaned and concentrated extracts were analyzed via RP-UPLC-photodiode array detection coupled with electrospray quadrupole time-of-flight tandem mass spectrometry (RP-UPLC-PDA-ESI-QTOF-MS and -MS/MS) on an Acquity UPLC system (Waters, DE), equipped with an Acquity UPLC PDA eλ detector (Waters, DE) and an ESI-QTOF-MS (maXis Impact, Bruker Daltonik GmbH, DE). Separation was done on an Ultra AQ C18 column 3 µm 150 × 2.1 mm including trident level 3 cartridge and filter applying a flow of 0.2 ml/min at 35 °C and 5 µl injection volume. The gradient composed of eluent A (water including 0.1% (v/v) formic acid) and eluent B (acetonitrile including 0.1% (v/v) formic acid) as follows: 0-10 min 100% A and 0% B; 11.5-12,5 min 40% A and 60% B; 14-15 min 100% A and 0% B. PDA-detection was done in a range between 210 and 800 nm with a sampling rate of 20 points/s and a resolution of 1.2 nm. MS analysis was performed in positive ionization mode at 200 °C dry temperature, 3 bar nebulizer, 4,000 V capillary voltage and a dry gas flow of 8 L/min. The MS method was adjusted to small molecule analysis (30–300 m*/z*) using a hexapole radio frequency (RF) voltage of 30 Vpp, a collision energy of 7 V, a funnel 1 and 2 RF of 80 Vpp, a pre-pulse storage time of 2 μs, a transfer time of 30.9 μs and a collision cell RF of 200 Vpp. MSMS detection was only applied for a few samples using auto MS/MS mode using CID with following parameters: absolute area threshold: 213 counts; exclusion activation: 3 spectra; exclusion release: 60 s; collision energy values (z = 1, 2, 3; isolation mass = 500; width = 8): 15, 10, 5 eV; collision energy values (z = 1, 2, 3; isolation mass = 1000; width = 10): 25, 20, 15 eV. The system was calibrated before each run with 10 mM sodium formate using linear calibration mode.

LC–MS data acquisition was controlled using the Hystar 3.2 software (Bruker Daltonik GmbH, DE). Data analysis was performed using the software package Bruker Compass DataAnalysis V4.4 SR1 (Bruker Daltonik GmbH, DE). The area of UV-absorbing organic acid peaks was quantified at 210 nm using the QuantAnalysis 4.4 or the DataAnalysis 4.4 software (Bruker Daltonik GmbH, DE). When available, authentic standards were used to confirm the annotations. Following MSMS analysis, fragmentation patterns were loaded in Metaboscape 4.0 (Bruker Daltonik GmbH, DE) for automated annotation of unknown patterns against Bruker MetaboBase and semi-quantitative comparison using MS1 data. The following settings were used: intensity threshold: 1,000 counts, minimum peak length: 7 spectra, minimum peak length recursive: 3 spectra, minimum of compounds for extraction: 2, no log mass calibration, primary ion: [M + H] + , expected ions: [M + Na] + and [M + K] + , EIC correlation: 0.8, mass range: 30-1000, RT range: 1-9.

#### Analysis of semi-polar metabolites

Semi-polar metabolites of sugar beet roots were analyzed in four sugar beet varieties (V1, V2, V5 and V6) at two timepoints; before and after 13 weeks of storage. The three biological replicates per variety were pooled at one timepoint. Extraction was done according to Petridis et al. (2016) [65] with minimal changes; A total of 100 mg of pooled, freeze-dried, and homogenized samples were extracted in one ml of 80% (v/v) methanol, twice. Combined extracts were concentrated 4 times by vacuum drying at room temperature. The extracts were cleaned on SPE cartridges prior to chromatographic analysis to reduce sugar contamination and to enrich semi-polar metabolites. For this purpose, Chromafix C18 (M) (Macherey–Nagel, DE) with Octadecyl-modified silica gel was used, one cartridge per sample. Cartridges were conditioned with 15 ml of methanol following equilibration with 15 ml of 2% (v/v) methanol including 0.5% (v/v) formic acid by applying mild vacuum. The sample was diluted to 2% (v/v) methanol in water and directly applied on the cartridge material. After washing with 15 ml of 2% (v/v) methanol including 0.5% (v/v) formic acid, semi-polar metabolites were eluted in 2 ml methanol including 0.5% (v/v) formic acid. Finally, samples were completely vacuum-dried, re-dissolved in 80 µl methanol and diluted to 80% (v/v) methanol including 0.1% (v/v) formic acid, which is the acidic solvent from the aqueous phase used in UPLC analysis.

Semi-polar metabolites were analyzed using RP-UPLC-PDA according to Petridis et al. (2016) [65]. The gradient composed of eluent A (water including 0.1% (v/v) formic acid) and eluent B (acetonitrile including 0.1% (v/v) formic acid) was as follows: 0-6 min 97% A and 3% B; 6-7.5 min 78% A and 22% B; 7.5-9.5 min 3% A and 97% B; 10-12 min 97% A and 3% B. PDA-detection was done in a range between 210 and 800 nm with a sampling rate of 20 pointss^−1^ and a resolution of 1.2 nm. Specific metabolites of interest were identified by RP-UPLC-PDA-ESI-QTOF-MS and tandem MS analysis using the same UPLC method as described above. The chromatographic system was the same as for organic acid analysis, but using different MS, tandem MS, and calibration methods. MS analysis was performed as described in Brauch et al. (2018) [66]. The analysis was performed in positive ionization mode at 200 °C dry temperature, 3 bar nebulizer, 4,000 V capillary voltage and a dry gas flow of 8 L/min. The MS method was adjusted to small molecule analysis (50–1,000 m*/z*) using a hexapole radio frequency (RF) voltage of 60 Vpp, a collision energy of 3 V, a funnel 1 and 2 RF of 300 Vpp, a pre-pulse storage time of 8 μs, a transfer time of 60 μs and a collision cell RF of 800 Vpp. MSMS detection was applied in auto MS/MS mode using CID with following parameters: absolute area threshold: 5000 counts; exclusion activation: 15 spectra; exclusion release: 60 s; collision energy values (z = 1, 2, 3; isolation mass = 500; width = 8): 15, 10, 5 eV; collision energy values (z = 1, 2, 3; isolation mass = 1000; width = 10): 25, 20, 15 eV. The system was calibrated before each run with 10 mM sodium formate using enhanced quadratic calibration mode. LC–MS data acquisition and analysis were similar to organic acids.

#### Statistics and plotting

Statistical analysis for raw metabolite data was done with pool samples (T0) consisting of 10 biological replicates. SigmaPlot 14 software [67] was used to calculate 1-way ANOVA “All Pairwise Multiple Comparison Procedures” applying “Tukey Test and Holm-Sidak method” for free amino acids and “Tukey Test” for organic acids. For semi-polar metabolites a statistical analysis was not possible since the samples were analyzed in pools. The free amino acid concentrations were scaled against the highest value of each amino acid from 0 to 1 to improve the comparison within the bar diagrams.

The computation of the principal component analysis (PCA) and heatmaps was done using R version 4.0.3 [68] specifically with the packages stats and gplots version 3.1.1 [69] respectively. The PCA was done on scaled data and visualized through the package ggplot2 version 3.3.5 [70]. The heatmaps were generated through the gplots function heatmap.2 using default parameters and including a row-based scaling.

### Transcriptomics and combinatorial omics visualization

Comparative transcriptomics between all varieties and timepoints is described in Madritsch et al. (2020) [17]. From this, the raw counts mapped against the reference genome (RefBeet-1.2.2) were used herein for the integrative visualization of metabolomics and transcriptomics data. The functional annotations (in terms of ENSEMBL IDs) for each transcript were downloaded from Ensembl Plants (release 51, accessed 05/21 [71]). Using the R package KEGGREST [72], the respective KEGG orthology (KO) number [73] and further information (GO terms [74] and the enzyme symbol) was retrieved. Transcripts that were assigned to the KEGG pathways belonging to the category “1.5 Amino acid metabolism” were selected for further analysis and were classified manually into either biosynthesis or degradation and metabolism for the focal amino acids. To further visualize the differential gene expression of genes involved in the above mentioned pathways for each selected amino acid, the computation of the sum of the shrunken log2 fold changes was done using R packages DESeq2 version 1.32.0 [75] and apeglm version 1.14.0 [76], whilst plotting was done using ggplot2 version 3.3.5 [70]. To estimate the impact of protein degradation on amino acid levels, out of the resulting significantly differentially expressed genes (DEG) list, those genes annotated with the GO category “proteolysis” (GO:0,006,508) were selected if |log2fc > 1| for at least one of the analyzed timepoints (T0 and T4) and were further filtered (adjusted *p*-value < 0.1). Visualization of up- and downregulated genes belonging to the respective biosynthesis (bvg00290, bvg00300, bvg00220, and bvg00400) or degradation and metabolism (bvg00250, bvg00260, bvg00270, bvg00280, bvg00310, bvg00330, bvg00340, bvg00350, bvg00360, and bvg00380) pathways for the focal amino acids was done using the R package ggplot2 version 3.3.5 [70]. Further combinatorial visualization of changes of selected pathways in both, transcriptomics, and metabolomics data, was done using the VANTED software version 2.8.1 [77] against selected pathways downloaded from MetaCrop 2.0 [78] as explicitly described [79]. 

## Supplementary Information

**Additional file 1:** **Supplemental Figure 1.** Free amino acid levels in sugar beet roots at T0. A: Scaled free amino acid concentrations. B: Free amino acid concentrations. In the left panel, well and badly storable varieties are compared. In the right panel, the three storage classes are compared (well, moderately, and badly storable varieties). Error bars represent standard deviation. From left to right varieties are shown in the following order: V6, V1, V3, V4, V2, V5. **Supplemental Figure 2.** Free amino acid concentrations in sugar beet roots at T0 comparing 10n (additional samples, left) and 3n (right) biological replicates. From left to right varieties are shown in the following order: V6, V1, V2, V5. **Supplemental Figure 3.** Scaled free amino acid contents in sugar beet roots at T4 comparing well and badly storable varieties (left) or well, moderately, and badly storable varieties (right). Error bars represent standard deviation. From left to right varieties are shown in the following order: V6, V1, V3, V4, V2, V5. **Supplemental Figure 4.** Loading plots of free amino acid concentrations prepared on respective PCA plots at T0, T4 and T0 - T4. **Supplemental Figure 5.** Comparison of sugar beet analysis and metabolite amounts in freeze dried root material. Amounts for the different metabolite classes were calculated as the average of total detected compounds per variety, per time point (e.g. Average (Sum 22 AA V1 T0; sum 22 AA V1 T2; sum 22 AA V6 T0; etc.). The resulting amounts are a rough estimation of the respective metabolite classes, which exclusively rely on detected and identified metabolites from this analysis. Undetected metabolites are neglected in this overview. Unidentified compounds were either skipped for organic acids or roughly quantified for semi-polar compounds. **Supplemental Figure 6.** Organic acid concentrations in sugar beet roots at T0 comparing 10n (left) and 3n (right) biological replicates. From left to right varieties are shown in the following order: V6, V1, V2, V5. **Supplemental Figure 7.** Pyroglutamic acid, glutamine, and glutamate concentrations in sugar beet roots at T0 - T4 comparing well and badly storable varieties. From left to right varieties are shown in the following order: V6, V1, V2, V5. **Supplemental Figure 8.** Organic acid contents in sugar beet roots comparing well and badly storable varieties at T0 - T4. Heat map is clustered by average. **Supplemental Figure 9.** Area per dry weight of semi-polar compounds detected at 280 nm in sugar beet roots that are upregulated during storage. Comparison of well (top, green) and badly (bottom, purple) storable varieties at T0 (color) and T4 (color pattern). Error bars represent 5%-error since biological replicates were pooled.

**Additional file 2:** **Supplemental Table 1.** Statistical evaluation of free amino acids in additional samples at T0 with ten biological replicates per variety. Sigma Plot, 1-way Anova per free amino acid using “All Pairwise Multiple Comparison Procedures (Tukey Test and Holm-Sidak method)”. Red: *P* < 0.001, blue: *P* < 0.01, yellow: *P* < 0.05. **Supplemental Table 2.** Statistical details of the evaluation of free amino acids in additional samples at T0 with ten biological replicates per each variety. All *P* < 0.050. Sigma Plot, 1-way Anova per free amino acid using “All Pairwise Multiple Comparison Procedures (Tukey Test and Holm-Sidak method)”. **Supplemental Table 3.** Statistical evaluation of organic acids in additional samples at T0 with ten biological replicates per variety. Sigma Plot, 1-way Anova per organic acid using “All Pairwise Multiple Comparison Procedures (Tukey Test)”. Red: *P* < 0.001, blue: *P* < 0.01, yellow: *P* < 0.05. **Supplemental Table 4.** Statistical details of the evaluation of organic acids in additional samples at T0 with ten biological replicates per each variety. All *P *< 0.050. Sigma Plot, 1-way Anova per organic acid using “All Pairwise Multiple Comparison Procedures (Tukey Test)”. **Supplemental Table 5.** 35 genes that fall under the GO category “proteolysis” (GO:0006508) and that are significantly differentially expressed between well and badly storable varieties. *DEGs present at both timepoints (T0 and T4). Log2 fold change values > |2| are marked in bold. (PDF 172 kb)

## Data Availability

Metabolite data that was generated and analyzed in this study is available on the e!DAL electronic data archive library [80] : http://dx.doi.org/10.5447/ipk/2022/14. Transcript data were deposited in the NCBI SRA database, accession number PRJNA610534 [17] .
