# Supplementary material for: Unraveling metabolic patterns and molecular mechanisms underlying storability in sugar beet
Source: BMC Plant Biol. 2022 Sep 9;22:430. doi: 10.1186/s12870-022-03784-6 (PMC9461268; doi:10.1186/s12870-022-03784-6)
Supplement: Supplementary file 1 — Additional file 1: Supplemental Figure 1. Free amino acid levels in sugar beet roots at T0. A: Scaled free amino acid concentrations. B: Free amino acid concentrations. In the left panel, well and badly storable varieties are compared. In the right panel, the three storage classes are compared (well, moderately, and badly storable varieties). Error bars represent standard deviation. From left to right varieties are shown in the following order: V6, V1, V3, V4, V2, V5. Supplemental Figure 2. Free amino acid concentrations in sugar beet roots at T0 comparing 10n (additional samples, left) and 3n (right) biological replicates. From left to right varieties are shown in the following order: V6, V1, V2, V5. Supplemental Figure 3. Scaled free amino acid contents in sugar beet roots at T4 comparing well and badly storable varieties (left) or well, moderately, and badly storable varieties (right). Error bars represent standard deviation. From left to right varieties are shown in the following order: V6, V1, V3, V4, V2, V5. Supplemental Figure 4. Loading plots of free amino acid concentrations prepared on respective PCA plots at T0, T4 and T0 - T4. Supplemental Figure 5. Comparison of sugar beet analysis and metabolite amounts in freeze dried root material. Amounts for the different metabolite classes were calculated as the average of total detected compounds per variety, per time point (e.g. Average (Sum 22 AA V1 T0; sum 22 AA V1 T2; sum 22 AA V6 T0; etc.). The resulting amounts are a rough estimation of the respective metabolite classes, which exclusively rely on detected and identified metabolites from this analysis. Undetected metabolites are neglected in this overview. Unidentified compounds were either skipped for organic acids or roughly quantified for semi-polar compounds. Supplemental Figure 6. Organic acid concentrations in sugar beet roots at T0 comparing 10n (left) and 3n (right) biological replicates. From left to right varieties are shown in the following order: [file 12870_2022_3784_MOESM1_ESM.pptx]

## Slide 1
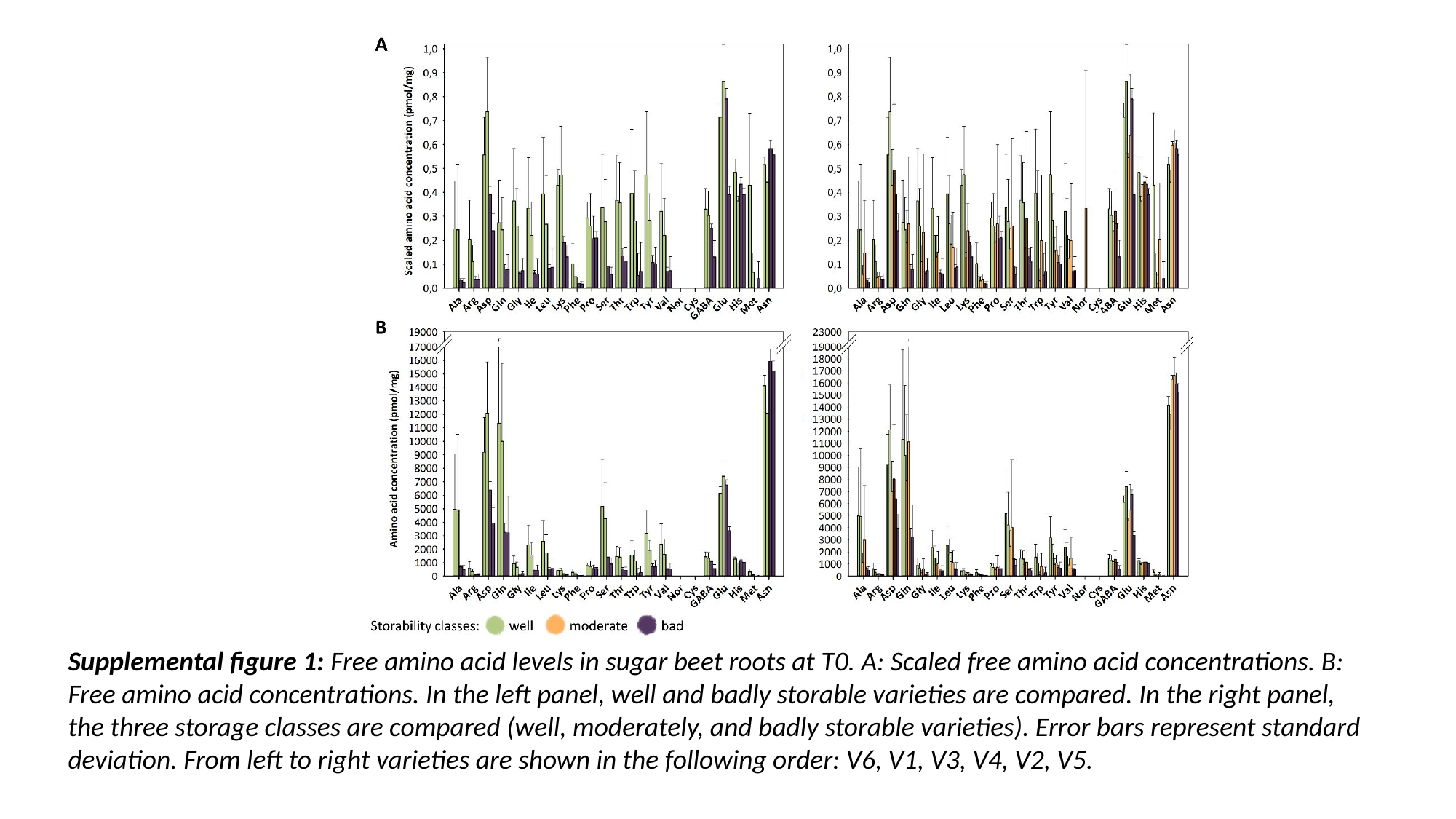

Supplemental figure 1: Free amino acid levels in sugar beet roots at T0. A: Scaled free amino acid concentrations. B: Free amino acid concentrations. In the left panel, well and badly storable varieties are compared. In the right panel, the three storage classes are compared (well, moderately, and badly storable varieties). Error bars represent standard deviation. From left to right varieties are shown in the following order: V6, V1, V3, V4, V2, V5.

## Slide 2
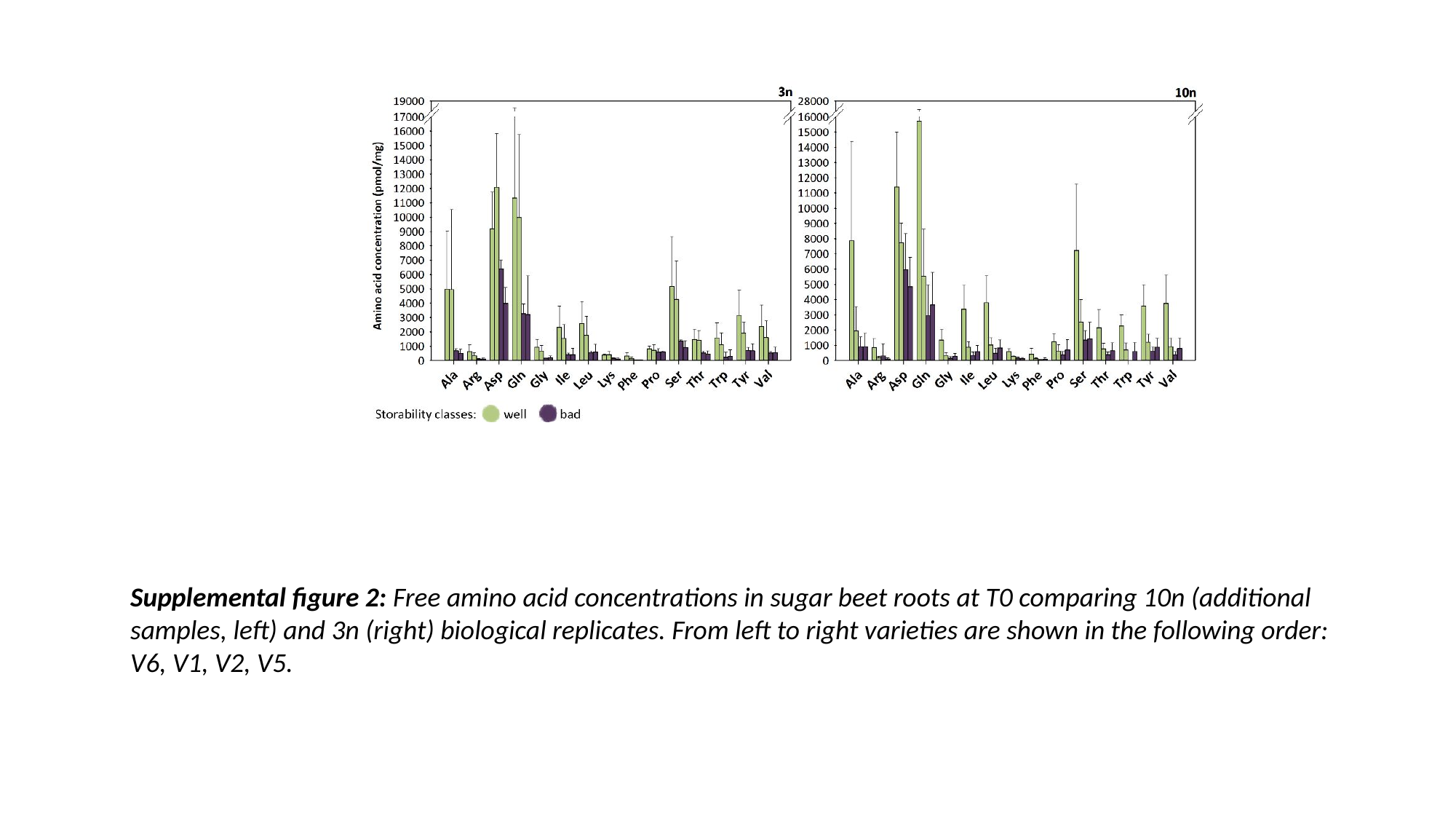

Supplemental figure 2: Free amino acid concentrations in sugar beet roots at T0 comparing 10n (additional samples, left) and 3n (right) biological replicates. From left to right varieties are shown in the following order: V6, V1, V2, V5.

## Slide 3
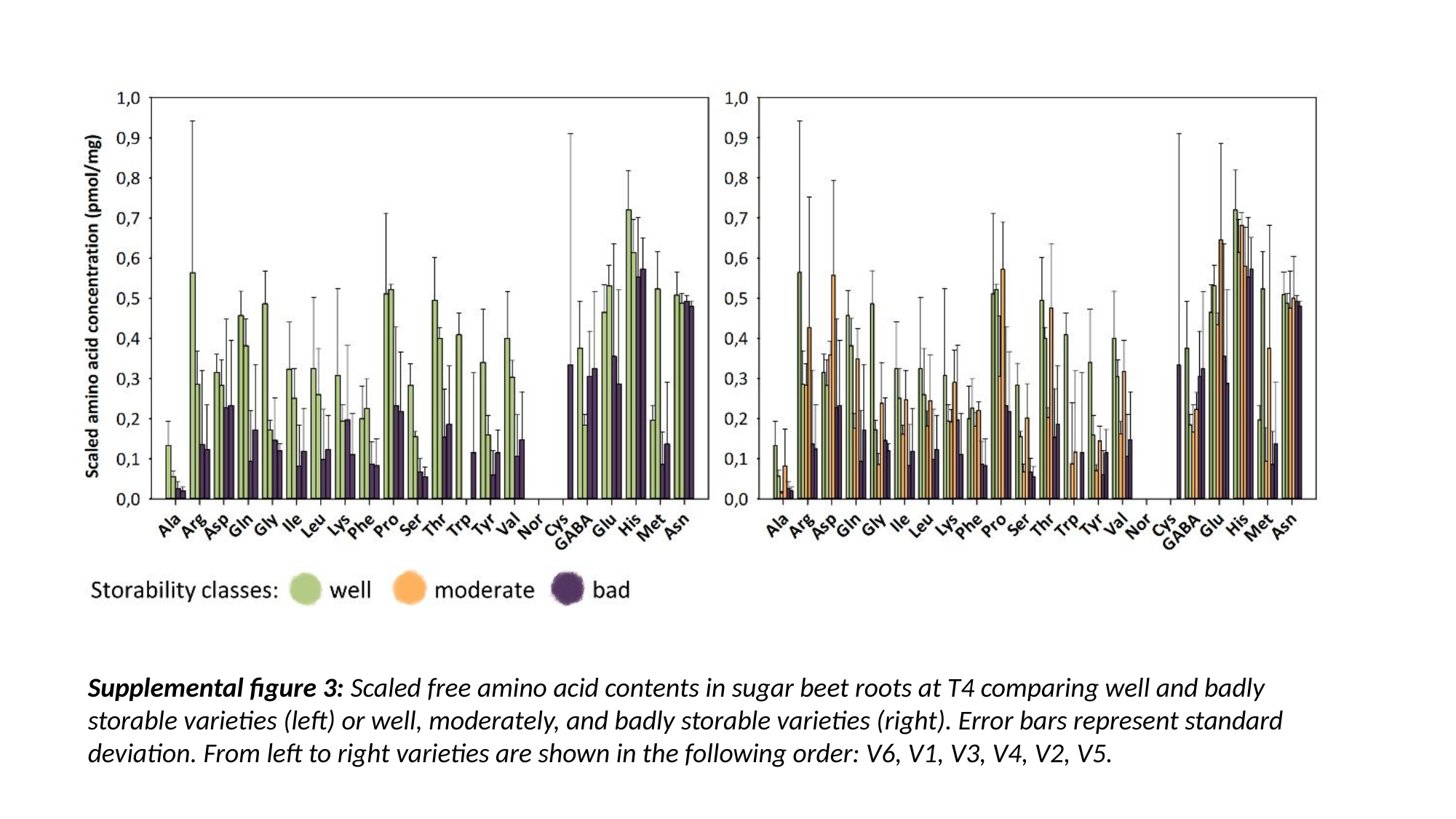

Supplemental figure 3: Scaled free amino acid contents in sugar beet roots at T4 comparing well and badly storable varieties (left) or well, moderately, and badly storable varieties (right). Error bars represent standard deviation. From left to right varieties are shown in the following order: V6, V1, V3, V4, V2, V5.

## Slide 4
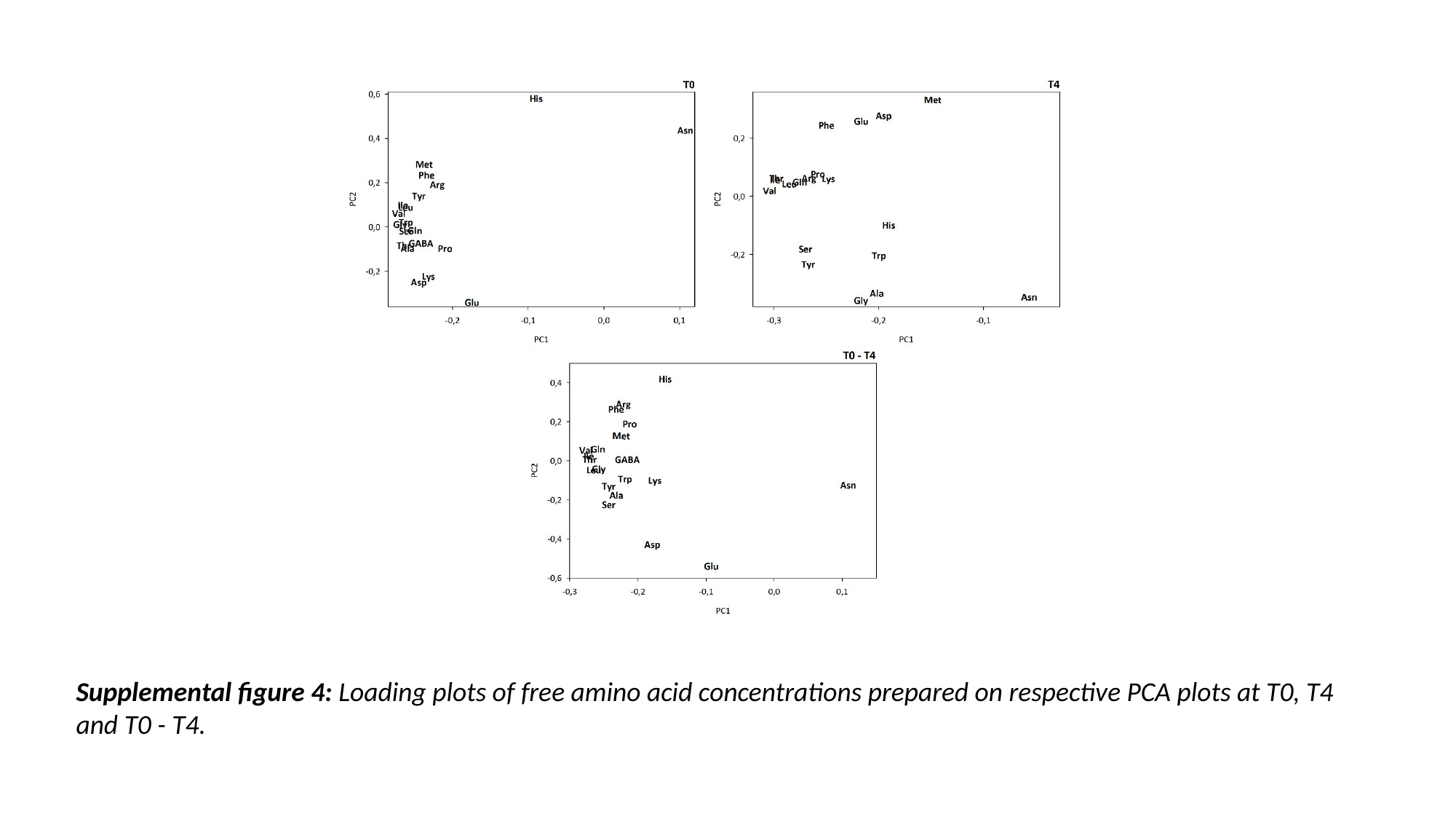

Supplemental figure 4: Loading plots of free amino acid concentrations prepared on respective PCA plots at T0, T4 and T0 - T4.

## Slide 5
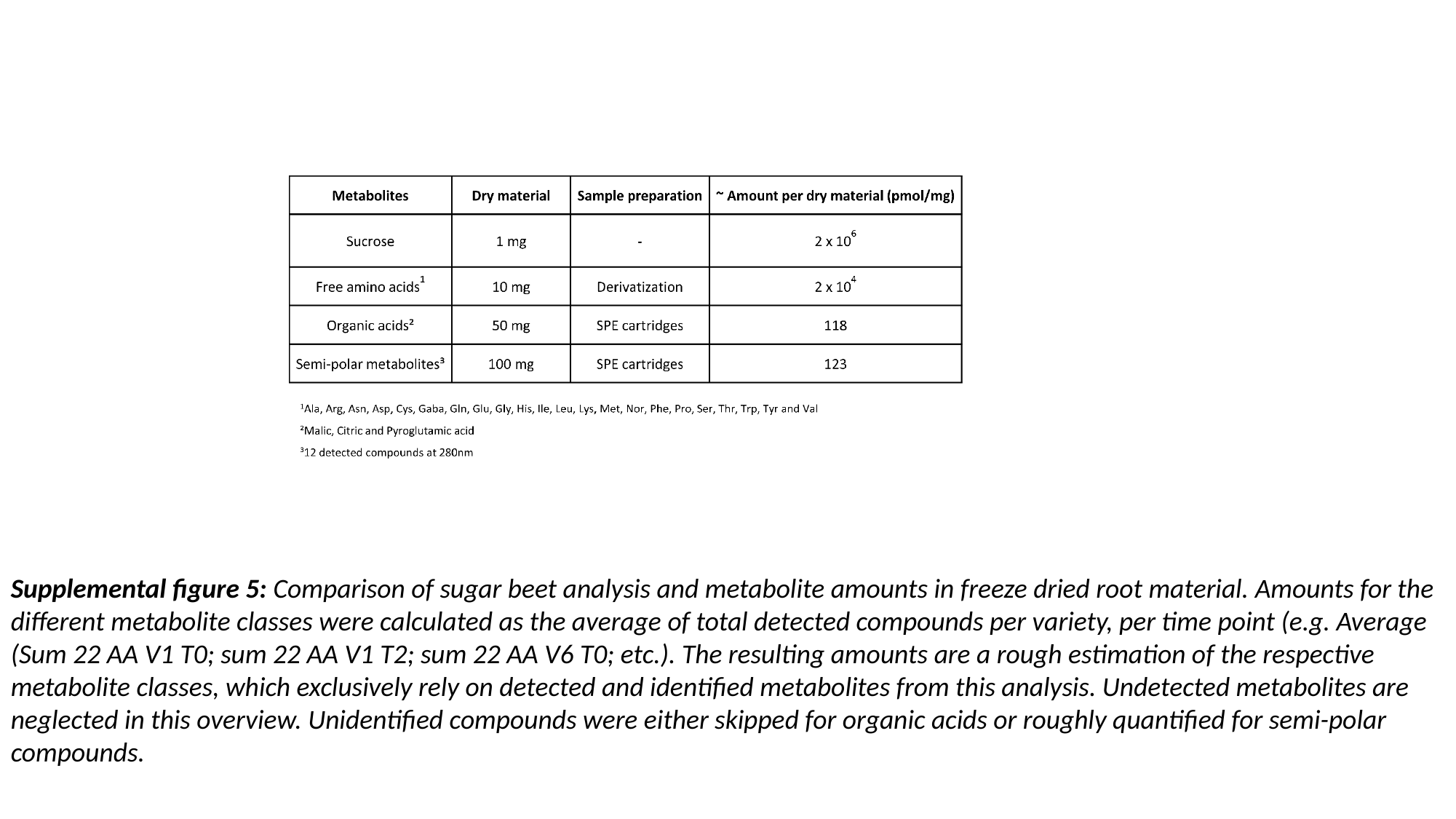

Supplemental figure 5: Comparison of sugar beet analysis and metabolite amounts in freeze dried root material. Amounts for the different metabolite classes were calculated as the average of total detected compounds per variety, per time point (e.g. Average (Sum 22 AA V1 T0; sum 22 AA V1 T2; sum 22 AA V6 T0; etc.). The resulting amounts are a rough estimation of the respective metabolite classes, which exclusively rely on detected and identified metabolites from this analysis. Undetected metabolites are neglected in this overview. Unidentified compounds were either skipped for organic acids or roughly quantified for semi-polar compounds.

## Slide 6
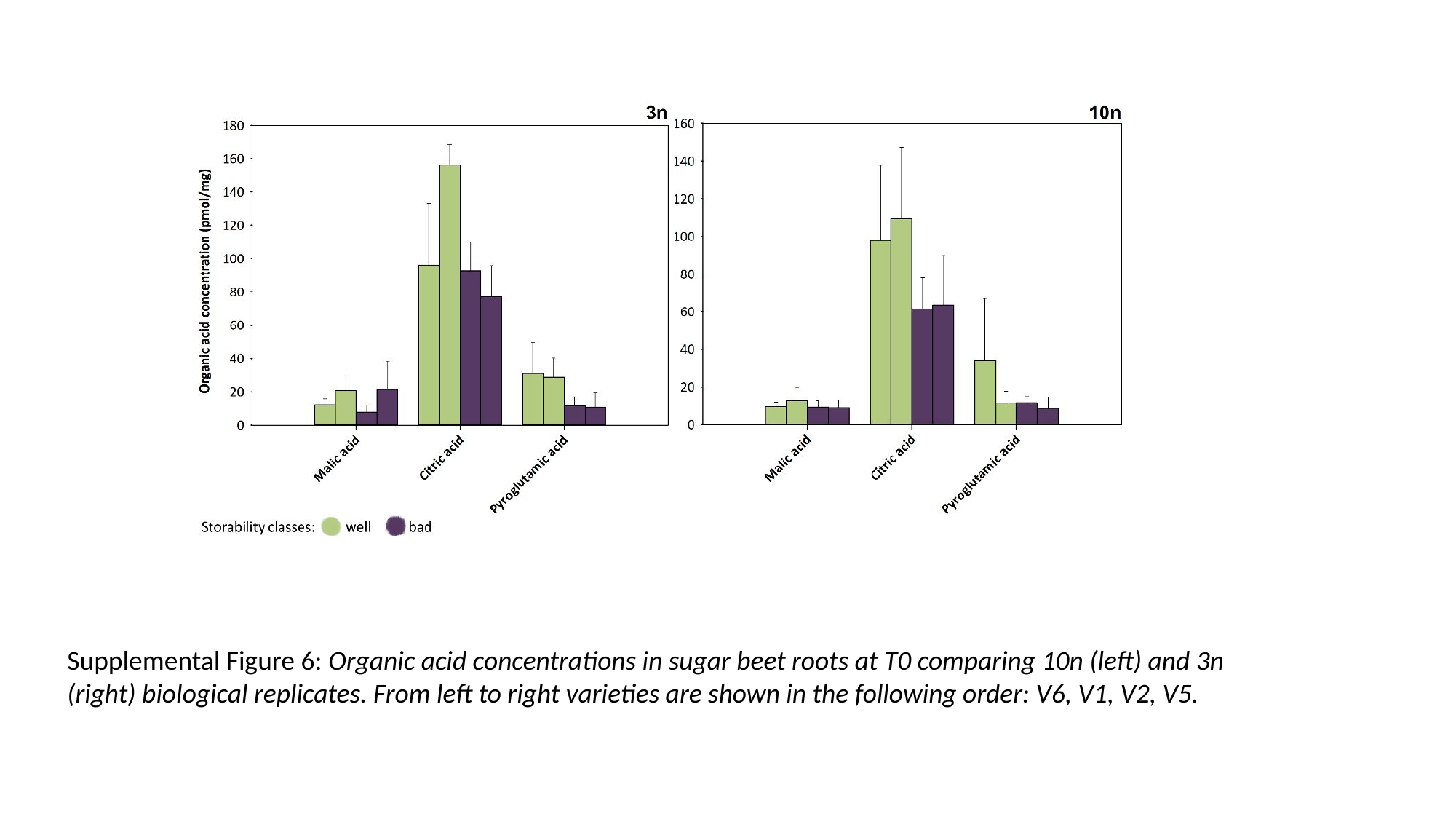

Supplemental Figure 6: Organic acid concentrations in sugar beet roots at T0 comparing 10n (left) and 3n (right) biological replicates. From left to right varieties are shown in the following order: V6, V1, V2, V5.

## Slide 7
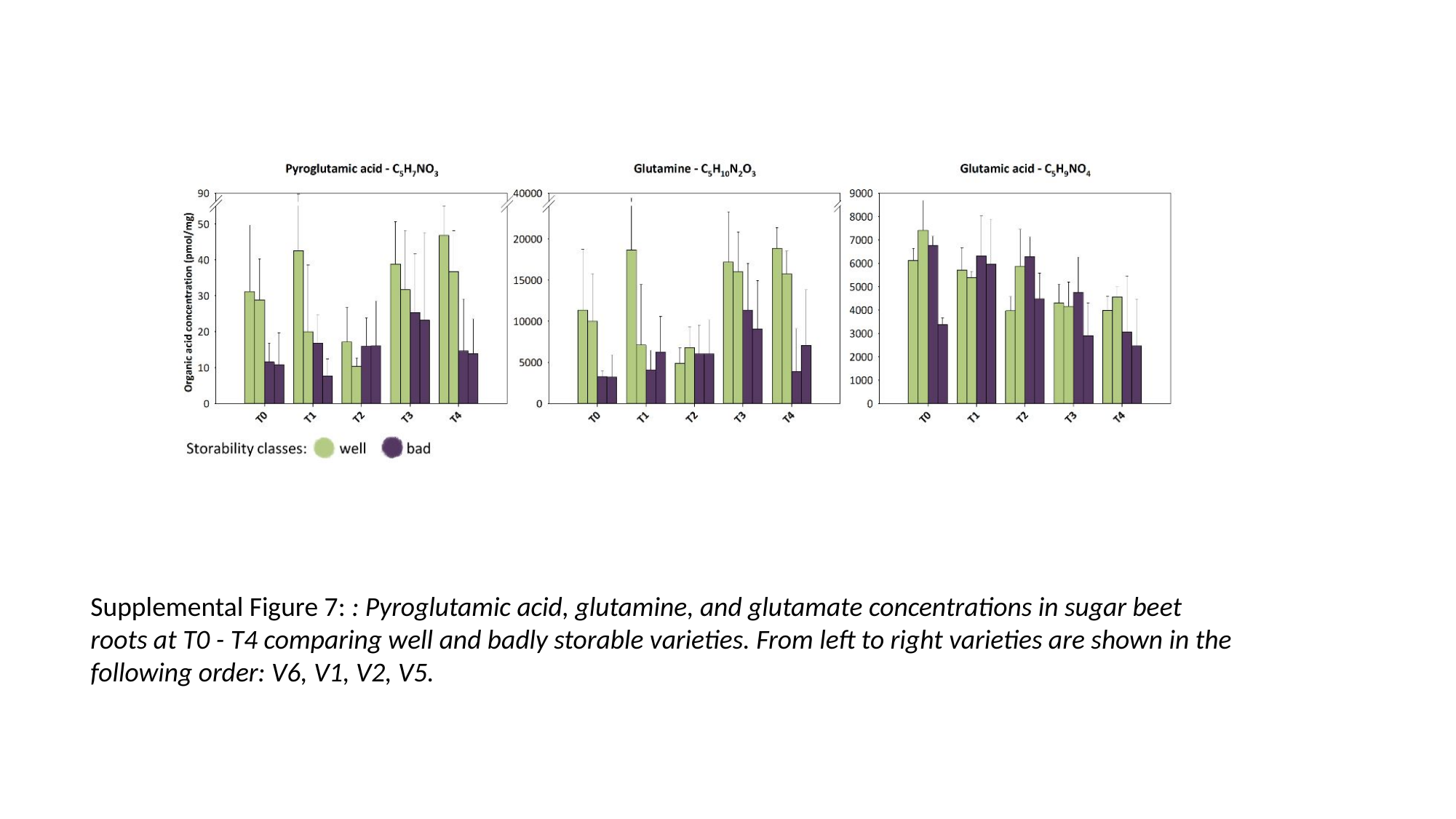

Supplemental Figure 7: : Pyroglutamic acid, glutamine, and glutamate concentrations in sugar beet roots at T0 - T4 comparing well and badly storable varieties. From left to right varieties are shown in the following order: V6, V1, V2, V5.

## Slide 8
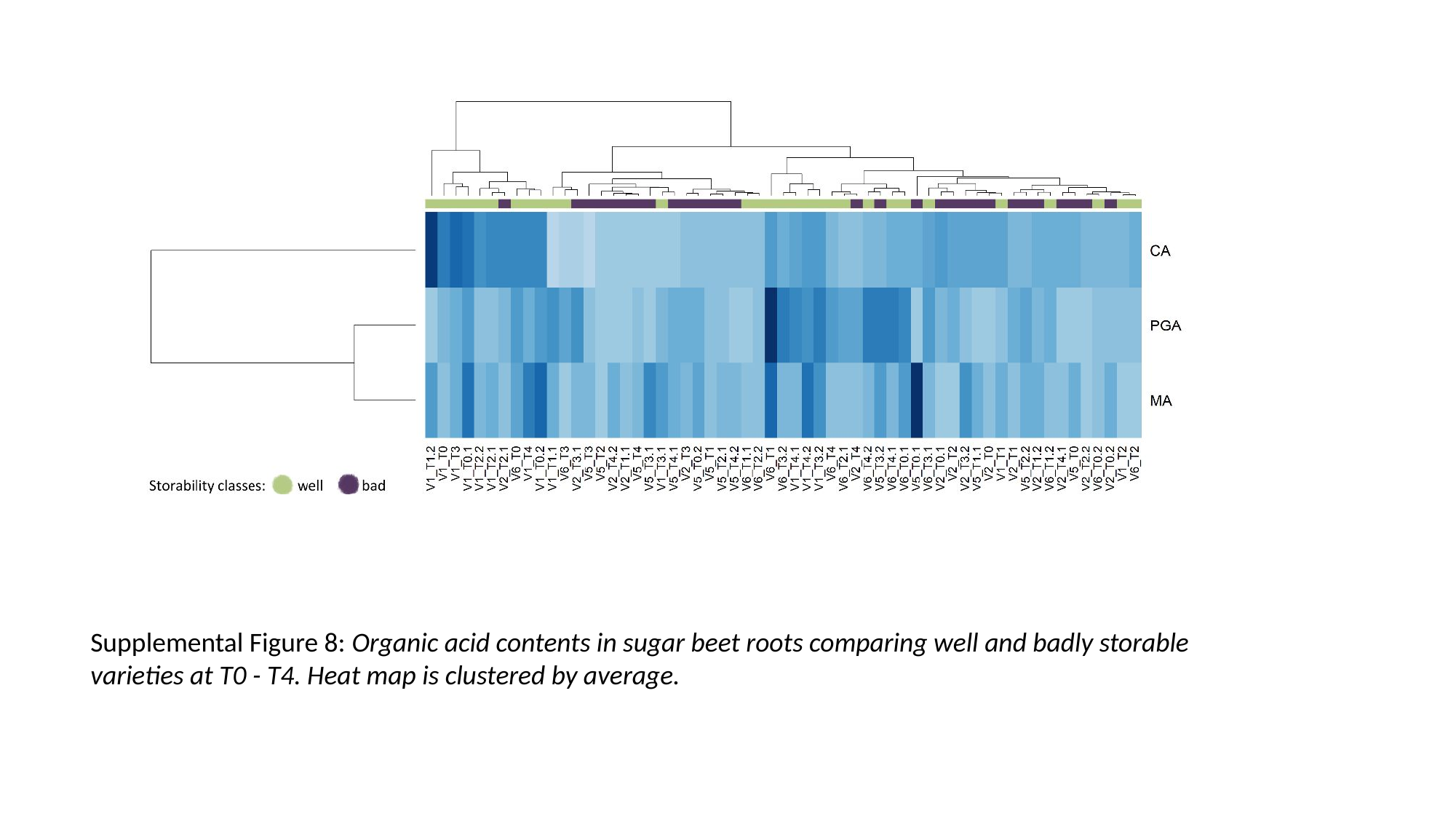

Supplemental Figure 8: Organic acid contents in sugar beet roots comparing well and badly storable varieties at T0 - T4. Heat map is clustered by average.

## Slide 9
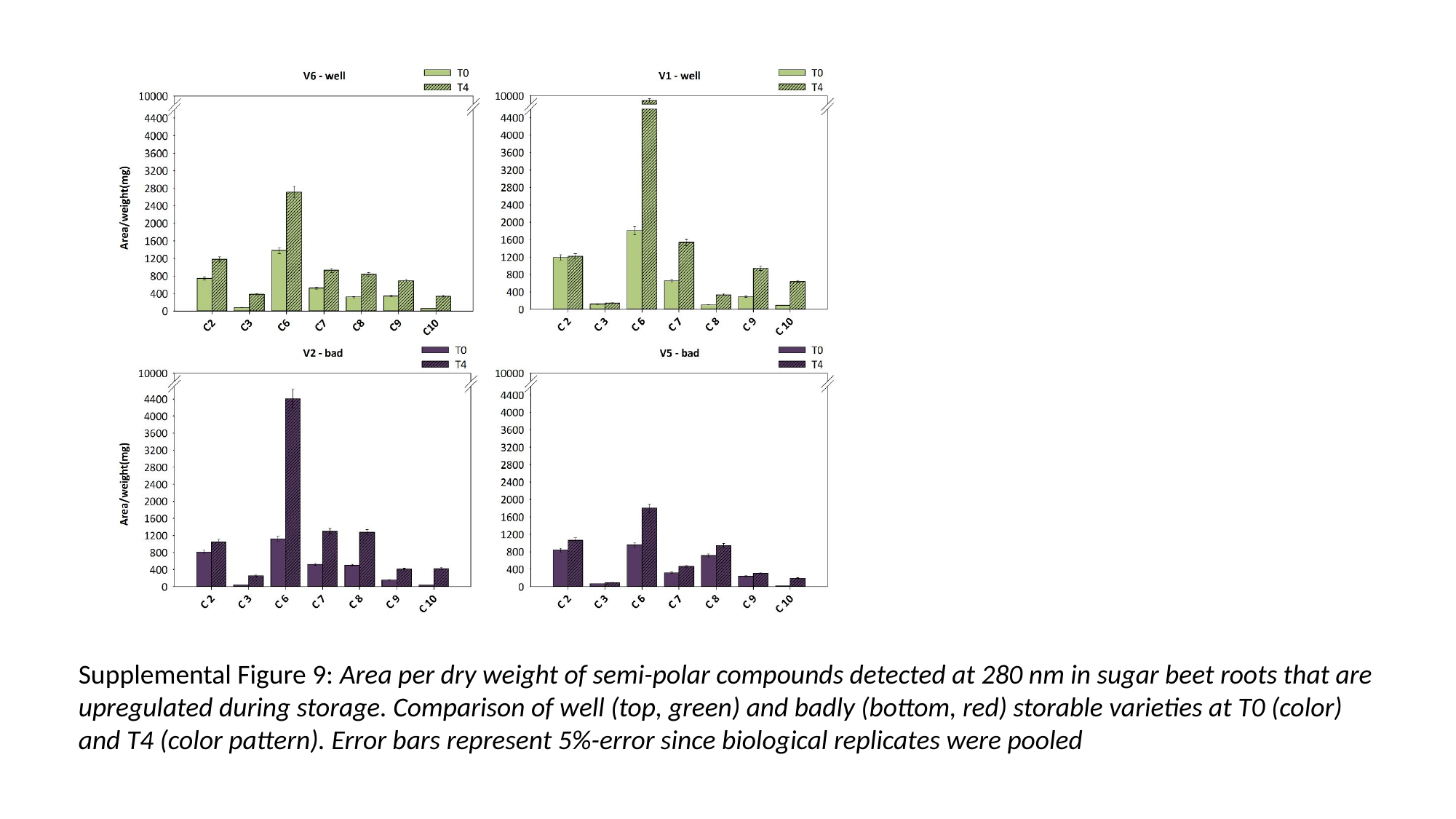

Supplemental Figure 9: Area per dry weight of semi-polar compounds detected at 280 nm in sugar beet roots that are upregulated during storage. Comparison of well (top, green) and badly (bottom, red) storable varieties at T0 (color) and T4 (color pattern). Error bars represent 5%-error since biological replicates were pooled
