# Supplementary material for: Unraveling metabolic patterns and molecular mechanisms underlying storability in sugar beet
Source: BMC Plant Biol. 2022 Sep 9;22:430. doi: 10.1186/s12870-022-03784-6 (PMC9461268; doi:10.1186/s12870-022-03784-6)
Supplement: Supplementary file 2 — Additional file 2: Supplemental Table 1. Statistical evaluation of free amino acids in additional samples at T0 with ten biological replicates per variety. Sigma Plot, 1-way Anova per free amino acid using “All Pairwise Multiple Comparison Procedures (Tukey Test and Holm-Sidak method)”. Red: P < 0.001, blue: P < 0.01, yellow: P < 0.05. Supplemental Table 2. Statistical details of the evaluation of free amino acids in additional samples at T0 with ten biological replicates per each variety. All P < 0.050. Sigma Plot, 1-way Anova per free amino acid using “All Pairwise Multiple Comparison Procedures (Tukey Test and Holm-Sidak method)”. Supplemental Table 3. Statistical evaluation of organic acids in additional samples at T0 with ten biological replicates per variety. Sigma Plot, 1-way Anova per organic acid using “All Pairwise Multiple Comparison Procedures (Tukey Test)”. Red: P < 0.001, blue: P < 0.01, yellow: P < 0.05. Supplemental Table 4. Statistical details of the evaluation of organic acids in additional samples at T0 with ten biological replicates per each variety. All P < 0.050. Sigma Plot, 1-way Anova per organic acid using “All Pairwise Multiple Comparison Procedures (Tukey Test)”. Supplemental Table 5. 35 genes that fall under the GO category “proteolysis” (GO:0006508) and that are significantly differentially expressed between well and badly storable varieties. *DEGs present at both timepoints (T0 and T4). Log2 fold change values > |2| are marked in bold. (PDF 172 kb) [file 12870_2022_3784_MOESM2_ESM.pdf]

SUPPLEMENTAL TABLES

Supplemental table 1: *Statistical evaluation of free amino acids in additional samples at T0 with ten biological replicates per Variety. Sigma Plot, 1-way Anova per free amino acid using "All Pairwise Multiple Comparison Procedures (Tukey Test and Holm-Sidak method)". Red: P < 0.001, blue: P < 0.01, yellow: P < 0.05.*

	P-value																				
**Comparison**	**Ala**	**Arg**	**Asn**	**Asp**	**Cys**	**Gaba**	**Gln**	**Glu**	**Gly**	**His**	**Ile**	**Leu**	**Lys**	**Met**	**Nor**	**Phe**	**Pro**	**Ser**	**Thr**	**Trp**	**Tyr**	**Val**
V6 vs. V5	<0.001	<0.001	0.046	<0.001	-	<0.001	0.002	<0.001	0.002	-	<0.001	0.001	<0.001	0.003	-	<0.001	-	<0.001	0.003	0.024	<0.001	<0.001
V6 vs. V2	<0.001	<0.001	<0.001	<0.001	-	0.012	<0.001	-	<0.001	-	<0.001	<0.001	<0.001		-	<0.001	<0.001	<0.001	<0.001	<0.001	<0.001	<0.001
V6 vs. V3	-	0.049	0.04	-	-	-	-	-	-	-	-	-	0.003	-	-	-	-	-	-	<0.001	-	-
V6 vs. V4	0.033	0.004	-	0.008	-	0.007	-	-	0.005	0.019	0.003	0.001	0.004	0.028	-	0.049	0.037	0.041	0.013	0.009	0.001	0.006
V6 vs. V1	-	-	-	0.013	-	-	-	-	0.015	-	0.034	0.021	-	-	-	-	0.048	-	0.041	-	-	0.019
V1 vs. V5	-	-	-	-	-	-	-	-	-	-	-	-	-	-	-	-	-	-	-	-	-	-
V1 vs. V2	-	0.049	-	-	-	-	-	-	-	0.008	-	-	-	-	-	-	-	-	-	0.049	-	-
V1 vs. V3	-	-	-	-	-	-	-	-	-	0.022	-	-	-	0.015	-	-	-	-	-	-	-	-
V1 vs. V4	-	-	-	-	-	-	-	-	-	-	-	-	-	-	-	-	-	-	-	-	-	-
V4 vs. V5	-	-	-	-	-	-	-	0.037	-	-	-	-	-	-	-	-	-	-	-	-	-	-
V4 vs. V2	-	-	0.02	-	-	-	-	-	-	<0.001	-	-	-	-	-	-	-	-	-	-	-	-
V4 vs. V3	-	-	-	0.039	-	0.007	-	-	-	<0.001	-	-	-	0.005	-	-	-	-	-	-	-	-
V3 vs. V5	-	-	-	<0.001	-	<0.001	0.008	0.022	-	0.006	-	-	-	<0.001	-	-	-	0.019	-	-	-	-
V3 vs. V2	-	-	-	<0.001	-	0.011	0.001	-	0.006	-	0.009	0.021	-	0.01	-	-	-	0.021	0.003	-	-	0.005
V5 vs. V2	-	-	-	-	-	-	-	<0.001	-	0.002	-	-	-	-	-	-	-	-	-	-	-	-

Supplemental table 2: *Statistical details of the evaluation of free amino acids in additional samples at T0 with ten biological replicates per each variety. All P < 0.050. Sigma Plot, 1-way Anova per free amino acid using "All Pairwise Multiple Comparison Procedures (Tukey Test and Holm-Sidak method)".*

Amino acids	Comparison	Diff of Ranks	q	P-value
**Ala**	V6 vs. V5	341	6.175	<0.001
	V6 vs. V2	318	5.758	<0.001
	V6 vs. V4	234	4.237	0.033
**Arg**	V6 vs. V2	408	7.388	<0.001
	V6 vs. V5	328	5.939	<0.001
	V6 vs. V4	284	5.142	0.004
	V6 vs. V3	223	4.038	0.049
	V1 vs. V2	223	4.038	0.049
**Asn**	V2 vs. V6	322	5.831	<0.001
	V2 vs. V4	247	4.472	0.02
	V3 vs. V6	229	4.147	0.04
	V5 vs. V6	225	4.074	0.046
**Asp**	V6 vs. V5	6572	6.066	<0.001
	V3 vs. V5	5923	5.467	<0.001
	V6 vs. V2	5442	5.023	<0.001
	V3 vs. V2	4793	4.424	<0.001
	V6 vs. V4	3869	3.571	0.008
	V6 vs. V1	3684	3.400	0.013
	V4 vs. V3	3220	2.972	0.039
**Gaba**	V3 vs. V5	314	5.686	<0.001
	V4 vs. V3	272	4.925	0.007
	V3 vs. V2	260	4.708	0.011
	V6 vs. V5	313	5.668	<0.001
	V6 vs. V4	271	4.907	0.007
	V6 vs. V2	259	4.690	0.012
**Gln**	V6 vs. V2	335	6.066	<0.001
	V6 vs. V5	297	5.378	0.002
	V3 vs. V2	307	5.559	0.001
	V3 vs. V5	269	4.871	0.008
**Glu**	V6 vs. V5	3200	4.998	<0.001
	V2 vs. V5	2829	4.419	<0.001
	V3 vs. V5	2111	3.298	0.022
	V4 vs. V5	1978	3.090	0.037
**Gly**	V6 vs. V2	415	7.514	<0.001
	V6 vs. V5	303	5.486	0.002
	V6 vs. V4	278	5.034	0.005
	V6 vs. V1	254	4.599	0.015
	V3 vs. V2	273	4.943	0.006
**His**	V2 vs. V4	359	6.500	<0.001
	V2 vs. V5	298	5.396	0.002
	V2 vs. V1	269	4.871	0.008
	V4 vs. V3	334	6.048	<0.001
	V3 vs. V5	273	4.943	0.006
	V3 vs. V1	244	4.418	0.022
	V6 vs. V4	248	4.491	0.019
**Ile**	V6 vs. V2	417	7.551	<0.001
	V6 vs. V5	333	6.030	<0.001
	V6 vs. V4	288	5.215	0.003
	V6 vs. V1	233	4.219	0.034
	V3 vs. V2	266	4.817	0.009
**Leu**	V6 vs. V2	410	7.424	<0.001
	V6 vs. V4	308	5.577	0.001
	V6 vs. V5	306	5.541	0.001
	V6 vs. V1	245	4.436	0.021
	V3 vs. V2	245	4.436	0.021
**Lys**	V6 vs. V5	363	6.573	<0.001
	V6 vs. V2	356	6.437	<0.001
	V6 vs. V3	287	5.188	0.003
	V6 vs. V4	284	5.142	0.004
**Met**	V3 vs. V5	330	5.966	<0.001
	V4 vs. V3	281	5.079	0.005
	V3 vs. V2	264	4.771	0.01
	V3 vs. V1	254	4.599	0.015
	V6 vs. V5	287	5.197	0.003
	V6 vs. V4	238	4.310	0.028
**Phe**	V6 vs. V2	385	6.971	<0.001
	V6 vs. V5	328	5.939	<0.001
	V6 vs. V4	223	4.038	0.049
**Pro**	V6 vs. V2	319	5.776	<0.001
	V6 vs. V4	231	4.183	0.037
	V6 vs. V1	224	4.056	0.048
**Ser**	V6 vs. V5	348	6.301	<0.001
	V6 vs. V2	345	6.247	<0.001
	V6 vs. V4	228	4.128	0.041
	V3 vs. V5	248	4.491	0.019
	V3 vs. V2	245	4.436	0.021
**Thr**	V6 vs. V2	387	7.007	<0.001
	V6 vs. V5	293	5.305	0.003
	V6 vs. V4	257	4.654	0.013
	V6 vs. V1	228	4.128	0.041
	V3 vs. V2	292	5.287	0.003
**Trp**	V6 vs. V2	409	7.406	<0.001
	V6 vs. V3	362	6.555	<0.001
	V6 vs. V4	265	4.798	0.009
	V6 vs. V5	242	4.382	0.024
	V1 vs. V2	223	4.038	0.049
**Tyr**	V6 vs. V2	399	7.225	<0.001
	V6 vs. V5	314	5.686	<0.001
	V6 vs. V4	305	5.523	0.001
**Val**	V6 vs V2	419	7.587	<0.001
	V6 vs V5	313	5.668	<0.001
	V6 vs V4	273	4.943	0.006
	V6 vs V1	248	4.491	0.019
	V3 vs V2	280	5.070	0.005

Supplemental table 3: *Statistical evaluation of organic acids in additional samples at T0 with ten biological replicates per Variety. Sigma Plot, 1-way Anova per organic acid using "All Pairwise Multiple Comparison Procedures (Tukey Test)". Red: P < 0.001, blue: P < 0.01, yellow: P < 0.05.*

	P-value		
**Comparison**	**MA**	**CA**	**PGA**
V6 vs. V5	-	-	<0.001
V6 vs. V2	-	-	0.019
V6 vs. V1	-	-	0.013
V1 vs. V5	-	0.041	-
V1 vs. V2	-	0.027	-
V5 vs. V2	-	-	-

Supplemental table 4: *Statistical details of the evaluation of organic acids in additional samples at T0 with ten biological replicates per each variety. All P< 0.050. Sigma Plot, 1-way Anova per organic acid using "All Pairwise Multiple Comparison Procedures (Tukey Test)".*

Organic acids	Comparison	Diff of Ranks	q	P-value
**CA**	V1 vs V2	146	3.949	0.027
	V1 vs V5	138	3.733	0.041
**PGA**	V6 vs V5	201	5.437	<0.001
	V6 vs V1	159	4.301	0.013
	V6 vs V2	152	4.112	0.019

Supplemental table 5: *35 genes that fall under the GO category "proteolysis" (GO:0006508) and that are significantly differentially expressed between well and badly storable varieties. *DEGs present at both timepoints (T0 and T4). Log2 fold change values > |2| are marked in bold*

	T0					T4						
	baseMean	log2FoldChange	lfcSE	pvalue	padj	baseMean	log2FoldChange	lfcSE	pvalue	padj	Gene ID	Gene description
BVRB_6g134130*	1482.115344	-0.658293515	0.292147137	0.002555184	0.019592441	4069.256861	-1.809416546	0.570869954	9.95266E-05	0.001442479	104895549	aspartyl protease family protein 1
BVRB_6g134140						266.3066554	**-2.205301464**	0.723766078	0.000115362	0.001631427	104895552	aspartyl protease family protein 1
BVRB_6g148390						117.161679	-1.092815395	0.446331498	0.001877395	0.014423004	104897293	serine carboxypeptidase-like 18
BVRB_6g149220*	57.51684177	**-2.530780174**	0.539204888	1.19355E-07	6.13254E-06	108.8419649	**-5.496155056**	1.307683374	8.17282E-07	2.77178E-05	104897419	aspartic proteinase CDR1-like
BVRB_6g151620*	278.8796092	0.833577274	0.146940736	1.49564E-09	1.34852E-07	370.1397537	1.80053028	0.232352578	8.87314E-16	4.59752E-13	104897646	probable thimet oligopeptidase
BVRB_5g100060*	519.296212	-1.067483312	0.486257729	0.001350082	0.012194151	507.4307974	-0.846905272	0.461482539	0.012240915	0.057383713	104892674	basic 7S globulin 2
BVRB_5g104400*	24.43789775	-0.844982141	0.406083407	0.002421684	0.018899172	169.4967845	**-5.711967963**	1.108019252	9.82681E-09	6.13042E-07	104892659	metalloendoproteinase 1-MMP
BVRB_5g108660						1851.628163	-1.175026583	0.957062259	0.012626797	0.058823087	104893167	basic 7S globulin
BVRB_5g116520*	108.8461668	-0.511301421	0.345139281	0.017342288	0.07963694	150.5153772	-1.143163118	0.55370517	0.004156635	0.025792987	104894226	protease Do-like 9
BVRB_5g116530	55.28387623	1.044873277	0.327641824	9.47018E-05	0.001557928						104894225	protease Do-like 9
BVRB_9g204140						470.7338718	**-3.197442881**	0.637433336	2.55687E-08	1.43223E-06	104902714	aspartic proteinase nepenthesin-1
BVRB_9g205630*	280.7716316	-0.44867402	0.295006842	0.022108887	0.094357667	21.01827038	**-3.335373439**	1.681996441	0.001014523	0.009045844	104902894	serine carboxypeptidase-like
BVRB_9g211890						307.430755	**-3.881756391**	0.808571491	6.78845E-08	3.37667E-06	104903717	basic 7S globulin
BVRB_7g159860						390.83781	-1.007389634	0.885429435	0.018738614	0.078107569	104898394	subtilisin-like protease SBT4.15
BVRB_7g169840						26.21470391	1.989275623	1.100406985	0.002628408	0.018417614	104900406	subtilisin-like protease SBT1.4
BVRB_8g180920						246.8583198	-1.069028426	0.512673235	0.004530711	0.027492305	104900491	probable cysteine protease RD21C
BVRB_8g181660*	842.1134262	**-3.630176511**	1.013878969	1.04719E-05	0.000260464	52.51070423	-0.586899057	1.394019151	1.03904E-05	0.000231004	104900552	zingipain-2
BVRB_8g181670*	712.2681412	**-3.940369977**	1.053186172	5.48014E-06	0.000150255	5.621515945	-0.326085451	0.681149644	0.002371776	0.017068187	104900551	zingipain-2
BVRB_8g181680*	209.3573731	**-3.678630191**	0.625825254	1.71003E-10	1.93192E-08	21.67300105	-0.661988409	1.957216817	0.000119662	0.001685846	104900553	zingipain-2
BVRB_8g181870*	16.44432118	**4.302555464**	0.951507216	2.42796E-07	1.10345E-05	3.660656783	0.338191777	0.688628201	0.007012829	0.038137112	104900574	serine carboxypeptidase-like 7
BVRB_8g191670	88.1635091	1.279305044	0.346564554	1.29218E-05	0.000306367						104901739	subtilisin-like protease SBT1.9
BVRB_1g002970*	96.57401221	-0.600628634	0.254096566	0.002329595	0.018430985	40.53628894	**-2.290633488**	0.934206583	0.000574918	0.005813226	104882986	aspartyl protease family protein 2
BVRB_1g013410*	4895.5967	0.462831357	0.165699075	0.001218123	0.011342942	5318.881324	1.008718226	0.24740947	8.90354E-06	0.000203527	104899002	hypothetical protein
BVRB_4g075000	2.148028883	**-2.011929824**	1.299521769	0.003795873	0.02629767						104890209	serine carboxypeptidase-like 20
BVRB_4g078200						388.9700145	-1.43215359	0.501964586	0.000378357	0.004188423	104890531	aspartic proteinase Asp1
BVRB_3g050270*	10.7321009	-0.198011376	0.384947637	0.011289885	0.058327963	32.5069333	**-5.829279025**	1.251733253	1.51372E-07	6.65928E-06	104888051	mitochondrial metalloendopeptidase OMA1
BVRB_1g019850*	249.0833895	-0.724284492	0.258889168	0.000520338	0.005917782	87.91346911	-1.229347223	0.425044348	0.000444552	0.004738415	104905471	subtilisin-like protease SBT4.14
BVRB_1g019870	16.76446907	-1.702474666	0.714197767	0.000604934	0.006651139						104905473	subtilisin-like protease SBT1.1
BVRB_3g069840						130.6250759	1.639129687	0.517311194	0.00012211	0.001710124	870846057	NA
BVRB_4g094890						369.0831134	-1.781181204	0.598405213	0.00018687	0.002418932	104907476	uncharacterized RNA-binding protein C1827.05c-like
BVRB_8g200730						35.29340859	**-4.666934326**	1.447656003	3.6301E-05	0.000643652	870843535	NA
BVRB_1g020870*	1227.679635	1.106172136	0.356670482	0.000118462	0.00183607	1564.858745	**2.353064804**	0.595101732	4.5017E-06	0.000115852	104905621	CO(2)-response secreted protease
BVRB_5g123180						1719.763055	-1.025028688	0.412386754	0.001939567	0.014757807	104907848	probable aspartyl protease At4g16563
BVRB_006890	7.524582834	1.182672553	0.812940974	0.004492779	0.029653294						104884217	cucumisin-like
BVRB_008150*	2010.014813	-1.763285201	0.377520539	1.46415E-07	7.20701E-06	5035.294932	**-5.479147778**	0.75884102	2.30917E-14	8.12698E-12	104884400	basic 7S globulin
